# PIRATE: A fast and scalable pangenomics toolbox for clustering diverged orthologues in bacteria

**DOI:** 10.1093/gigascience/giz119

**Published:** 2019-10-09

**Authors:** Sion C Bayliss, Harry A Thorpe, Nicola M Coyle, Samuel K Sheppard, Edward J Feil

**Affiliations:** The Milner Centre for Evolution, Department of Biology and Biochemistry, Claverton Down, University of Bath, Bath BA2 7AY, UK

**Keywords:** microbial genomics, pangenomics, next-generation sequencing, bioinformatics

## Abstract

**Background:**

Cataloguing the distribution of genes within natural bacterial populations is essential for understanding evolutionary processes and the genetic basis of adaptation. Advances in whole genome sequencing technologies have led to a vast expansion in the amount of bacterial genomes deposited in public databases. There is a pressing need for software solutions which are able to cluster, catalogue and characterise genes, or other features, in increasingly large genomic datasets.

**Results:**

Here we present a pangenomics toolbox, PIRATE (Pangenome Iterative Refinement and Threshold Evaluation), which identifies and classifies orthologous gene families in bacterial pangenomes over a wide range of sequence similarity thresholds. PIRATE builds upon recent scalable software developments to allow for the rapid interrogation of thousands of isolates. PIRATE clusters genes (or other annotated features) over a wide range of amino acid or nucleotide identity thresholds and uses the clustering information to rapidly identify paralogous gene families and putative fission/fusion events. Furthermore, PIRATE orders the pangenome using a directed graph, provides a measure of allelic variation, and estimates sequence divergence for each gene family.

**Conclusions:**

We demonstrate that PIRATE scales linearly with both number of samples and computation resources, allowing for analysis of large genomic datasets, and compares favorably to other popular tools. PIRATE provides a robust framework for analysing bacterial pangenomes, from largely clonal to panmictic species.

## Background

For most bacteria the complement of genes for a given species is far greater than the number of genes in any single strain. Comprising core genes shared by all individuals in a species and accessory genes that are variously present or absent, the pangenome represents a pool of genetic variation that underlies the enormous phenotypic variation observed in many bacterial species. Through horizontal gene transfer, bacteria can acquire genes from this pangenome pool that bestow important traits such as virulence or antimicrobial resistance [1].

Over the past decade, advances in whole-genome sequencing technologies and bioinformatic analyses have allowed the cataloguing of genes and intergenic regions that make up the pangenomes of many species [2–9].

Current approaches define genes on the basis of strict sequence identity thresholds [2,3, 7,8], e-value cut-offs [5,6], and bit score ratios [4]. However, genes accrue variation at different rates under the influence of positive and purifying selection [10]. Therefore, it is difficult to define a single identity threshold beyond which genes cease to belong to the same family. Relaxed thresholds risk over-clustering of related gene families, whilst conservative thresholds risk over-splitting, by misclassifying highly divergent alleles of the same gene into multiple clusters. Over-splitting is likely to be especially problematic in vertically acquired core genes that have undergone strong diversifying selection or horizontally acquired accessory genes from multiple source populations that share a distant common ancestor. The impact of over- and under-clustering is relevant to consider in the context of downstream research applications. Under-clustering (or over-splitting) can create a misleading impression of pangenome diversity and composition when considering how much gene diversity exists in the accessory genome [9]. However, for a study identifying genetic determinants associated with a phenotype, such as antibiotic resistance, core and accessory allelic variation that has been misclassified as additional accessory genes may have little to no impact because the causative genes in question may still be correctly identified.

In order to address these considerations we have created the Pangenome Iterative Refinement and Threshold Evaluation (PIRATE) toolbox, which evaluates and classifies genetic diversity within the pangenome. PIRATE provides the means to create pangenomes from any annotated features (e.g. coding sequence, transfer RNA, ribosomal RNA) over a user-defined range of amino acid or nucleotide identity thresholds. PIRATE provides measures of sequence divergence and allelic diversity within the sample. PIRATE also categorizes paralogs into duplication and/or fission loci, loci disrupted by an insertion, deletion, or nonsense mutation. A consistent nomenclature is applied to allow for the user to identify gene clusters that are the product of duplication or fission events, providing additional context on both methodological and evolutionary gene provenance. This rapid, scalable method allows for a comprehensive overview of gene content and allelic diversity within the pangenome.

## Methods

### Pangenome construction

**Figure 1.**
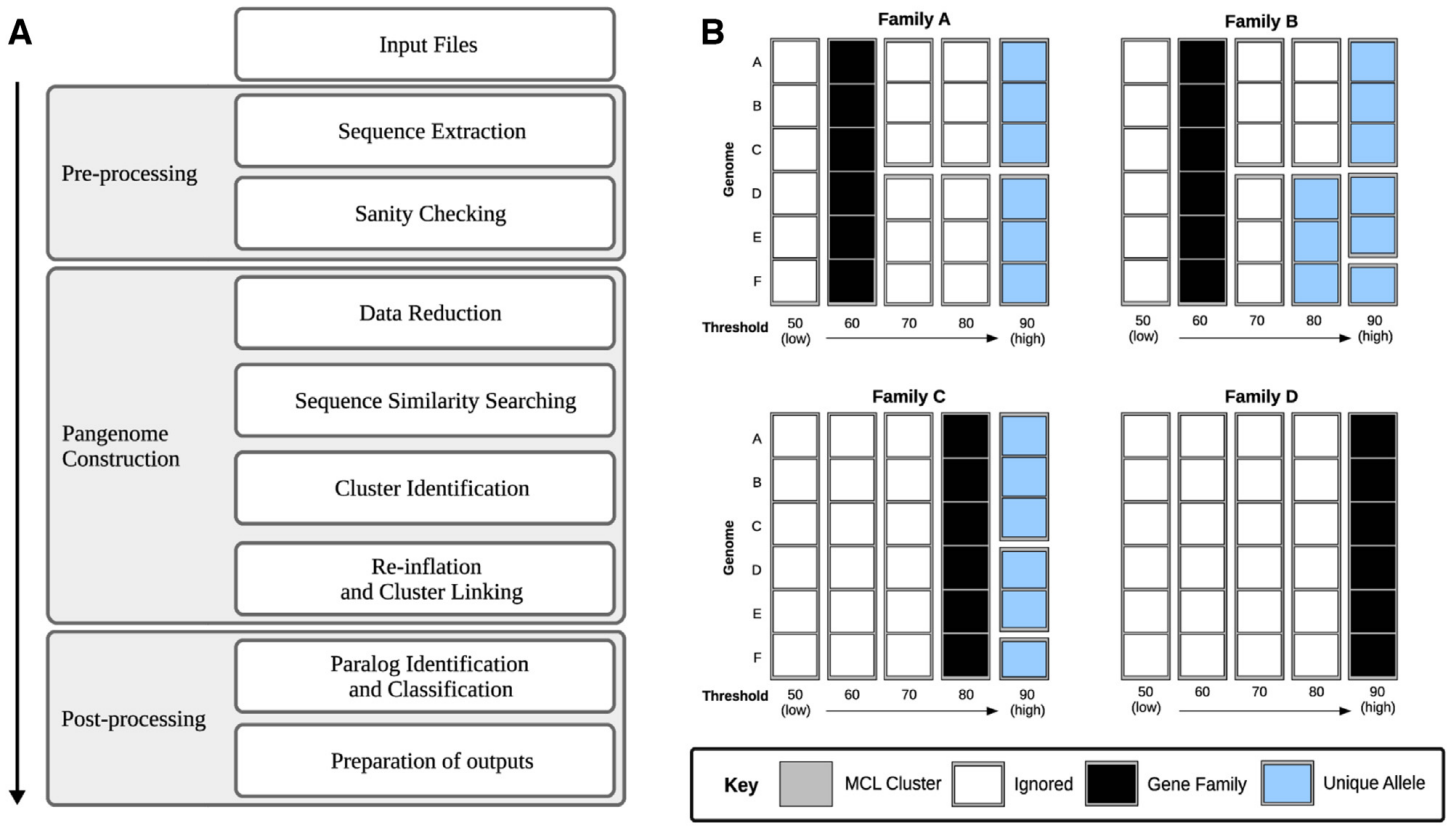
(A) Flow chart denoting a simplified workflow. (B) Example cluster classification. Blocks represent sequences from unique genomes. Grey blocks represent MCL clusters at various percentage identity cut-offs. Black squares indicate a “gene family” cluster, the lowest percentage identity threshold from the MCL clustering. Blue squares represent “unique alleles,” MCL clusters at higher percentage identity thresholds with unique combinations of sequences (at the higher threshold at which they are observed together). White squares represent redundant MCL clusters; these are not present in the PIRATE output.

The PIRATE pipeline has been summarized as a schematic in Fig. 1A. The input is a set of GFF3 files. Feature sequences are filtered and the dataset is reduced by iterative clustering using CD-HIT [2,11]. The longest sequence from each CD-HIT cluster is used as a representative for sequence similarity searching (BLAST/DIAMOND) [12,13]. The normalized bit scores of the resulting all-vs-all comparisons are clustered using MCL after removing hits that fall below a relaxed threshold of percentage identity (default: 50%) [14]. A default MCL inflation value of 2 was identified as appropriate for intra-species clustering by the present study and previous authors [2]. A larger inflation value may be appropriate for inter-species comparisons and can be modified as appropriate. The initial clustering at this lower bounds threshold is used to define putative “gene families” (Fig. 1B). Initial designations may not represent the final outputs because families containing paralogs may be subsequently split during the paralog splitting step. MCL clustering is repeated over a range of user-specified percentage identity thresholds (default 50–95% amino acid identity, increments of 5). Unique MCL clusters at higher thresholds are used to identify “unique alleles” (Fig. 1B). Loci may be shared between multiple unique alleles (MCL clusters) at different percentage identity thresholds (e.g., Fig. 1B - Family B). PIRATE uses the highest threshold at which a “unique allele” is observed to define the shared percentage identity in the resulting outputs.

### Paralog classification

Clusters that contain >1 sequence per individual genome are putative paralogs and undergo an additional post-processing step (Supplementary Figure 6). All loci are clustered on the basis of sequence length (98% similar) using CD-HIT. Homology between representative loci is established using all-vs-all BLAST. Loci with no significant overlaps are considered putative fission loci and are compared against a reference sequence (the longest sequence in the gene family), which is considered the most “complete” version of the gene. All combinations of putative fission loci are compared to the reference in order to find the combination that gives the most parsimonious coverage of the reference sequence. This combination locus is classified as a “fission locus” that may have formed via gene disruption (e.g., insertion, deletion, or nonsense mutation). Any locus that overlaps with all other loci or is not a part of a fission cluster is considered a duplication. The process is iterated until all loci have been classified.

### Cluster splitting

After paralog classification, fission loci are treated as a single locus. Gene families that contain genomes with multiple loci, after accounting for fission loci, potentially represent 2 or more related gene families that have been over-clustered. In these cases the gene family is checked against the presence of MCL clusters (unique alleles), which contain a single copy of the loci in all constituent genomes (Supplementary Figure 6). These alleles are thereafter considered separate gene families with nomenclature denoting their shared provenance (e.g. g0001_1, g0001_2).

### Post-processing

Syntenic connections between gene families in their source genomes are used to create a pangenome graph. Parsimonious paths between gene families contained in the same number of genomes are used to identify co-localized gene families. This information is used to order the resulting tabular pangenome file on syntenic blocks of genes in descending order of number of genomes that those blocks were present in. Gene-by-gene alignments are produced using MAFFT to generate a core gene alignment [15]. Installing the relevant dependencies in R allows for PIRATE to produce a pdf containing descriptive figures.

A number of supplementary tools are provided to extract, align, and subset sequences and to compare and visualize outputs. To facilitate integration with existing pipeline, scripts have been provided to convert the outputs of PIRATE into common formats, which allows for them to be used as inputs to software used for downstream analysis, such as the PanX user interface, SCOARY, Microreact, or Phandango [6,16–18]. A full description of the methodology and comparative benchmarks has been provided in the supplementary information (Supplementary Information).

## Results and Discussion

### Benchmarking and comparison to other tools

The performance of PIRATE was assessed on a range of parameters related to its scalable application to large numbers of bacterial genomes. Three bacterial species were selected for comparison, *Campylobacter jejuni*, *S. aureus*, and *Escherichia coli*, representing both a range of pangenome sizes (small, medium, and large, respectively) and guanine-cytosine content (30.4%, 32.7%, and 50.6%, respectively)(Supplementary Table 2). The scripts used to perform these analyses are available from the GigaDB repository associated with the publication [19]. The settings used for each tool have been detailed in Supplementary Table 3. Memory usage and wall time were found to scale approximately linearly with increasing numbers of isolates, and the amount of memory and time per sample was consistent (Supplementary Figures 1 and 3). PIRATE has been extensively parallelized, and the availability of additional cores was found to significantly reduce runtime (Supplementary Figure 2).

**Figure 2.**
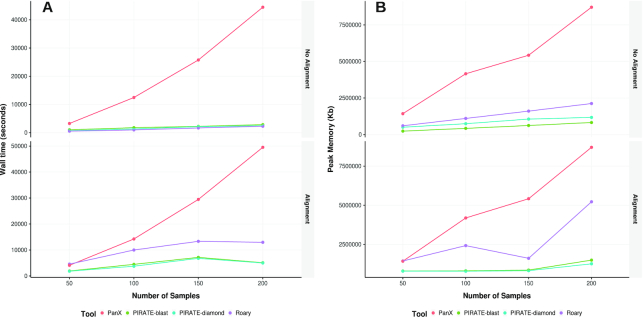
Benchmarking of PIRATE against Roary and PanX. Wall time (seconds) and peak memory usage (KB) were recorded for each tool run on a dataset of 50, 100, 150, 200, and 250 complete *Staphylococcus aureus* genomes from the RefSeq database with and without gene-by-gene alignment.

A range of tools have been developed for constructing bacterial pangenomes. For comparison, we chose 2 of the most widely used packages, Roary and PanX [2,6]. These tools have some similarities to PIRATE that facilitate comparison; all 3 tools share similar clustering workflows (BLAST/DIAMOND, MCL) and require annotated genomes as input. Differences in methodology lie primarily in the post-processing of clusters: Roary uses a single percentage identity threshold for MCL clustering and separates paralogs based upon their neighboring genes, and PanX splits paralogous genes using an alignment/tree-based method rather than the CD-HIT–BLAST approach used by PIRATE. Each of the 3 tools were applied to subsets of 50, 100, 150, 200, and 250 *S. aureus* complete genomes downloaded from the RefSeq database (Supplementary Table 2), for comparisons on the same hardware using 8 cores [20]. It should be noted that both PIRATE and Roary include post-processing of paralogs in the comparison without alignment or phylogenetic tree reconstruction, producing a complete output. PanX does not do this because alignment, followed by tree building, is a necessary step in paralog identification in this pipeline. Therefore, analyses were run with and without gene-by-gene alignment in order to make unbiased comparisons. Execution time and memory usage per sample were recorded (Fig. 2). To aid comparison PanX was used with the -dmdc flag, which batches input genomes and clusters per batch and subsequently merges the batches. Without this option the runtime of PanX scales quadratically and is inappropriate for larger datasets and comparison to the other tools.

The execution time of Roary and PIRATE scaled in an approximately linear manner with increasing number of samples (Fig. 2A). Roary and PIRATE were faster than PanX at all time points without gene-by-gene alignment. The execution time of PIRATE using DIAMOND was comparable to that of Roary without gene-by gene alignment (Fig. 2A, top panel). Roary completed marginally quicker than PIRATE using BLAST without gene-by-gene alignment at all sample sizes. When gene-by-gene alignment was applied both Roary and PIRATE scaled sub-linearly with number of samples; however, PIRATE using DIAMOND or BLAST completed substantially faster than either Roary or PanX (Fig. 2A, bottom panel). PIRATE exhibited lower memory usage than the other tools tested, scaling sub-linearly with number of samples (Fig. 2B). In conclusion, PIRATE compared favourably in both execution time and memory usage, and these metrics suggest that PIRATE can be flexibly applied to large datasets on routinely available hardware.

### Application to real datasets

PIRATE has been applied to 3 real datasets: *Staphylococcus aureus, Prochlorococcus marinus*, and *Pseudomonas. S. aureus*, a gram-positive human commensal and opportunistic pathogen, was used as a benchmarking dataset for comparison to other tools. Additionally, PIRATE was applied to a further 2 datasets to highlight its application to large or diverse pangenomes. PIRATE was applied to 45 draft genomes of *P. marinus*, a marine cyanobacteria with extremely diverse gene complement, and a collection of 497 complete genomes of assorted *Pseudomonas* species, a genus of gram-negative Gammaproteobacteria that have highly variable genome sizes.

### Staphylococcus aureus

**Figure 3.**
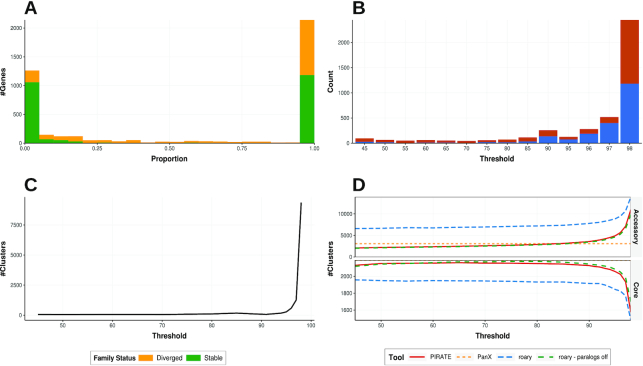
Descriptive figures of the pangenome of 253 complete *Staphylococcusaureus* genomes inferred using PIRATE. PIRATE was run with default parameters over a range of amino acid identity values (45–98%). (A) The proportion of genomes in which gene families are found, indicating stable gene families (green) with a single allele at 98% amino acid identity, and diverged with >1 allele (yellow). (B) The minimum amino acid percentage identity cut-off at which all loci were present per gene family (core = blue, accessory = red). (C) The number of unique alleles at each amino acid percentage threshold. A unique allele is characterized as the highest percentage identity threshold at which a unique sub-cluster of isolates from a single gene family was identified by MCL. (D) Comparison of core and accessory gene/allele estimates for PIRATE (red), PanX (orange), Roary (blue), and Roary with paralog splitting switched off (green). The estimates represent “allelic” variation reported by PIRATE in contrast to “gene content” variation reported by the other tools. PanX provided a single estimate of core and accessory genome content because it has no analogous command to -s in PIRATE or -i in Roary to allow comparison. Core gene families are characterized as being present in >95% of genomes. All tools were run on default parameters. Roary was run over a range of thresholds matching those used for PIRATE with and without paralog splitting (-s).

PIRATE was applied to 253 complete *S. aureus* genomes downloaded from the RefSeq database (accessed 8 November 2018) (Supplementary Table 2) [21]. PIRATE was run on default settings over a wide range of amino acid percentage identity thresholds (45, 50, 60, 65, 70, 75, 80, 85, 90, 91–99 in increments of 1%) (Supplementary Table 2). The pangenome of *S. aureus* comprised 4,250 gene families of which 2,433 (57.25%) were classified as core (>95% genomes) and 1,817 (42.75%) as accessory (Fig. 3A). Gene families with an average copy number >1.25 loci per genome after paralog classification were excluded from further analysis (178 gene families [4.19%]) because direct comparison between high copy number or potentially over-clustered families is problematic. Of the remaining 4,072 gene families, 740 (18.17%) clustered at thresholds of <95% percentage identity. At these thresholds a significantly different number of “divergent” gene families were observed (χ^2^ test *P*-value = < 0.0001) between core and accessory genomes; 21.83% of accessory genes (383/1,754) clustered at <95% homology compared with only 15.40% of core genes (357/2,318) (Fig. 3B). A possible explanation for this is that the accessory genes may have been horizontally acquired and therefore may be from diverse genetic backgrounds with different evolutionary histories.

PIRATE can quickly be used to identify genes with both highly conserved or divergent sequence similarity or variable copy number. The biological ramifications of these genes will vary between applications. For example the core “accessory regulator” *agr* locus exhibited a range of sequence identity clustering thresholds; *agrA* clusters at 91%, *agrB* and *agrC* at 65%, and *agrD* at 45% amino acid identity, each with a copy number of 1. We identified that another gene, *arlR*, which is known to interact with the *agr* locus, has a similarly low amino acid similarity of 45%, perhaps implying that the linked genes have undergone similar patterns of diversifying selection. This example highlights how diversification may lead to over-splitting of genes if only a single sequence identity threshold were used, even if this threshold were applicable to the vast majority of genes in the pangenome. Expansion of families of mobile genetic elements or individual genes within the population can also be identified from the outputs. For example, the transposase for IS256, known to play a role in biofilm formation and resistance to various antimicrobial agents, is present in 35 genomes and has a conserved amino acid sequence (<2% divergence) but a variable copy number of between 1 and 32 copies within the genomes in which it is present. Using these data it is possible to identify the strains that have an increased copy number of IS256.

A steep increase in the number of unique clusters per threshold (allelic diversity) of the sample was observed at thresholds >90% (Fig. 3C). At these thresholds allelic variation will begin to influence the identification of gene families in analogous tools [2,7–8]. In addition to this metric, PIRATE identifies the highest threshold at which all loci in a gene family cluster together. This value can be used to estimate the sequence similarity threshold at which alleles are classified as “genes” by analogous tools (before paralog processing) and therefore allows for evaluation of the influence of this choice on core and accessory genome sizes (Fig. 3D). For comparison, Roary and PanX were applied to the *S. aureus* dataset (default settings). Roary was run at a range of percentage identity thresholds matching those used by PIRATE (-i option) to facilitate comparison. Paralog splitting in Roary was also switched off (-s option) to assess the influence of paralog splitting on the resulting pangenome size estimates. The number of core and accessory genes (<95% isolates) estimated by both tools was compared with those estimated using PIRATE (Fig. 3D). All tools give similar estimates of the number of core genes (PIRATE = 2,141, PanX = 2,191, Roary [-i 45] = 1,959, Roary no paralogs [-i 45] = 2,118). However, estimates of the number of accessory genes were divergent (PIRATE = 2,190, PanX = 3,097, Roary [-i 45] = 6,620, Roary no paralogs [-i 45] = 2,046).

For the *S. aureus* collection the estimated number of core genes remains fairly constant at thresholds <90% and decreases sharply at thresholds >95% (Fig. 3D). This suggests that the majority of the *S. aureus* core genome would be reconstructed by tools that identify genes as clusters of sequences with >10% amino acid sequence similarity. However, the impact of more conservative thresholds on the accessory genome is pronounced. A moderate increase in the number of alleles misidentified as low-frequency genes was observed at thresholds <90% followed by a sharp increase at thresholds >90%. This suggests that, even at low identity thresholds, allelic diversity in highly divergent genes inflates the number of clusters incorrectly identified as “accessory” genes when only a single homology threshold is used. This effect is likely to be more pronounced in organisms with large accessory genomes owing to a higher number of diversified gene families in the accessory genome.

The outputs from the 3 tools were compared to identify the differences in the gene clusters that they produced. Loci not present in all outputs, due to tool-specific input sequence filters, were removed. PIRATE produced 4,247 clusters, PanX 5,193, and Roary 10,454. The clusters were compared in a pairwise manner between tools, and the numbers of matching clusters were identified (Supplementary Figure 7). Clusters were considered matching when they contained the same loci and were ±5% the size (number of loci) of the query cluster. The relaxed cluster size threshold (±5%) was applied to allow for minor discrepancies between the clusterings that were unlikely to significantly affect the interpretation of results. The majority of clusters matched between PIRATE and PanX (PanX: PIRATE = 3,515/5,193 [67.69%], PIRATE-PanX = 3,456/4,247 [81.38%]). Many of the mismatches occurred in the accessory or intermediate pangenome. The greater number of PIRATE clusters identified in the PanX output was likely due to the less aggressive paralog-splitting algorithm and co-clustering of truncated genes (fission/fusion genes) used by PIRATE. The majority (∼70%) of PIRATE and PanX clusters were found in the output of Roary (PanX: Roary = 3,736/5,193 [71.94%], PIRATE: Roary = 2,979/4,247 [70.14%]), suggesting that a large proportion of core genes were found by all tools. The smallest number of matching clusters (∼25%) were between Roary and the clusters identified by the other tools (Roary: PanX = 3,029/10,454 [28.97%], Roary: PIRATE = 2,419/10,454 [23.14%]), and most of these mismatches were observed in accessory clusters. We would suggest that this is due to the aggressive splitting of paralogous genes in Roary, the implications of which have been documented by previous authors [9].

These results suggest that there was a large intersection in the core gene clusters and, to a lesser extent, accessory clusters, of the 3 tools studied. However, the tools varied in the identification of shared clusters in the intermediate and accessory pangenomes. This difference was more pronounced in accessory genes identified by Roary than between PIRATE and PanX. The vast majority of the differences in clustering between tools is most likely due to the different paralog-splitting methodologies used. Other variations in methodology, such as the “divide-and-conquer” strategy used by PanX or the co-clustering of fission/fusion genes by PIRATE, may also contribute to this variation to a lesser extent. The close approximation by PIRATE of accessory content variation in Roary without paralog splitting suggests that PIRATE can be used to provide accurate estimates of pangenome composition for analogous tools before paralog splitting.

### *Pseudomonas* species

**Figure 4.**
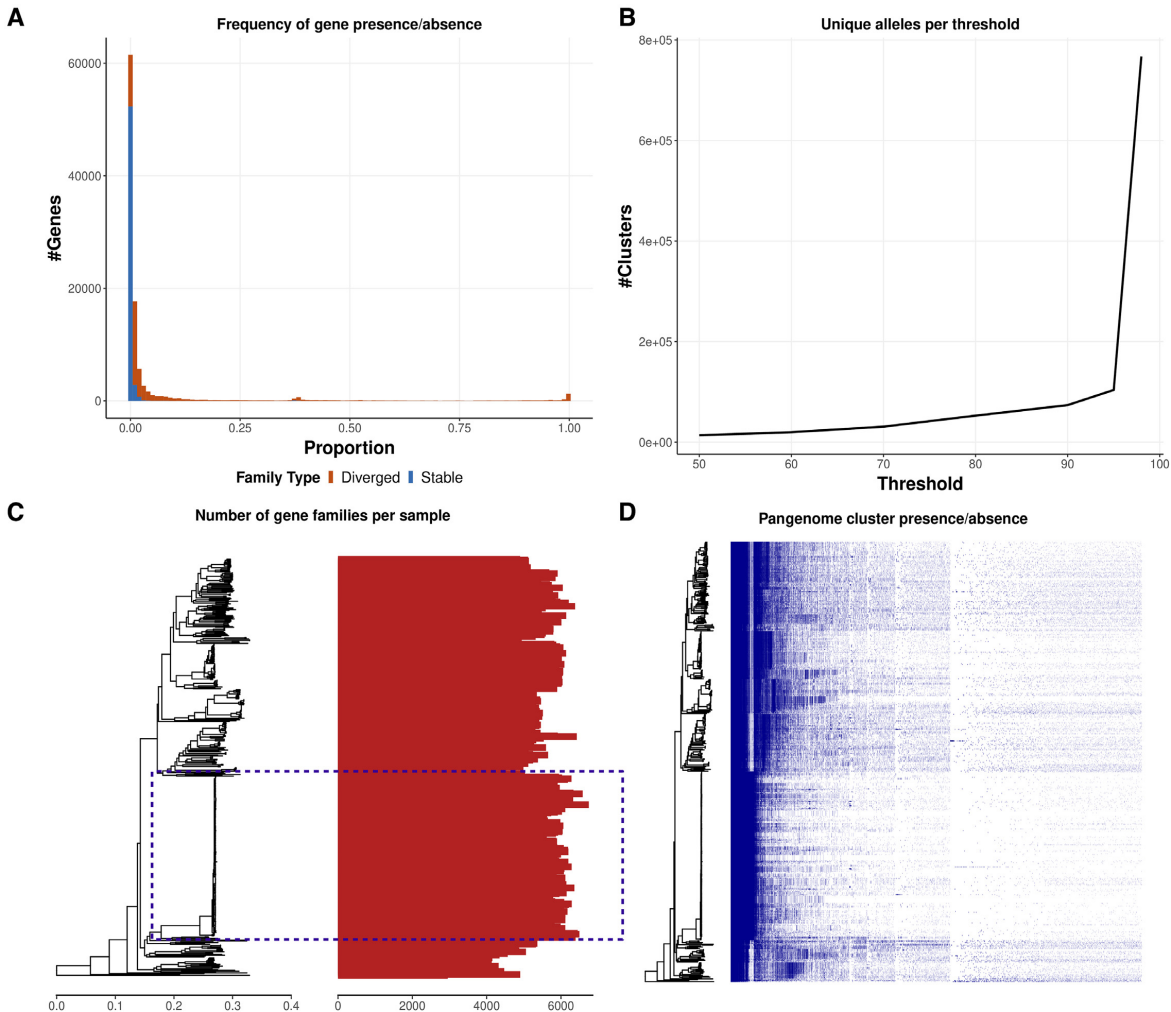
Summary figures of the pangenome of 496 *Pseudomonas* complete genomes. PIRATE was run on default parameters with an MCL inflation value of 6 and an HSP query length threshold of 0.9. (A) The proportion of genomes in which gene families are present. Gene families are considered stable (blue) when they have only a single allele at 98% amino acid identity, and diverged (red) when they have >1 allele. (B) The number of unique alleles at each amino acid percentage threshold. A unique allele is characterized as the highest percentage identity threshold at which a unique sub-cluster of isolates from a single gene family was identified by MCL. (C) The number of gene families per isolate ordered alongside the phylogenetic tree. (D) Shared gene presence per isolate ordered alongside the phylogenetic tree. Gene family presence is indicated by a blue block per column. Phylogenetic trees were generated from a core gene alignment from PIRATE and constructed using rapidnj [22].

PIRATE was applied to a dataset of 496 complete genomes of assorted, uncharacterized *Pseudomonas* species from the NCBI database (Supplementary Table 2) [21]. The pangenome of the *Pseudomonas* collection was reconstructed, including gene-by-gene sequence alignment, in 188,216 s (52.3 h) using 12 threads, an MCL inflation value of 6, and a high-scoring pair (HSP) query length threshold of 0.9. The pangenome comprised 2,858,820 loci clustered into 102,425 gene clusters of which 1,841 (1.8%) were considered core (present in >95% of isolates) (Fig. 4A). An increase in the frequency of genes present in ∼40% of the isolates corresponded to “lineage core” genes from an over-represented lineage (Fig. 4C, dotted blue box). The number of unique alleles per genome increased at percentage identity thresholds >70%, most likely representing inter-species/lineage divergence, and increased sharply at thresholds >94–95% (Fig. 4B). This increase was consistent with the sharp increase of intra-species allelic diversity observed in other datasets investigated within this study (Fig. 4B). *Pseudomonas* had an extremely variable genome size (4.7–11 Mb), which was reflected in the number of genes present per isolate (Fig. 4C). There was an observable relationship between genetic relatedness and number of genes per isolate with considerable within-lineage variation. This is most clearly observable in the most numerous lineage present in the collection (Fig. 4C, dotted blue box), which contained between 5,000 and 7,000 genes per isolate. Whilst there were a large number of lineage core genes present in *Pseudomonas* species, there were also a number of promiscuous genes intermittently present or absent across all *Pseudomonas* genomes analysed (Fig. 4D).

### Prochlorococcus marinus

**Figure 5.**
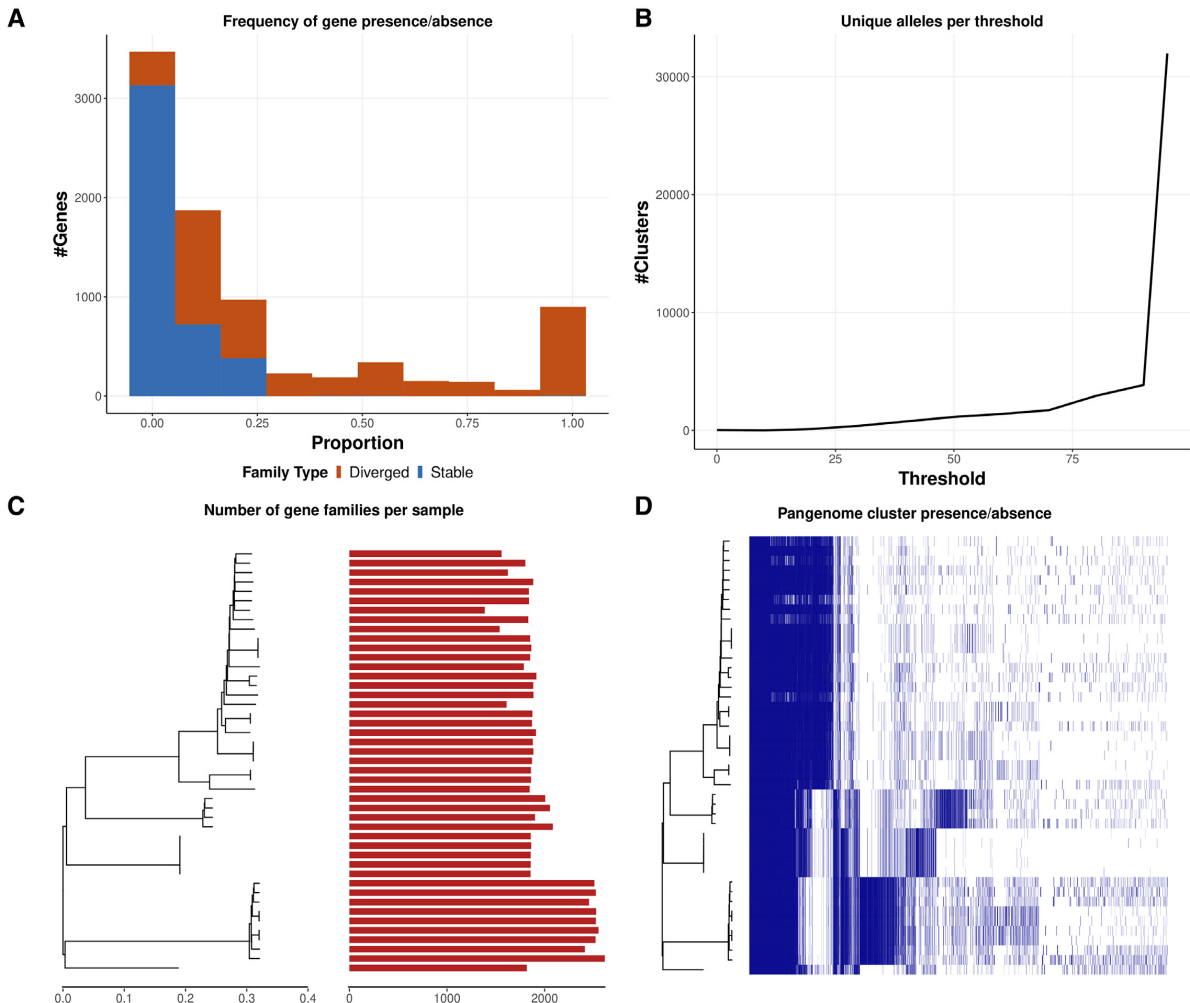
Summary figures of the pangenome of 45 *Prochlorococcus marinus* draft genomes. PIRATE was run on default parameters with an MCL inflation value of 6, an HSP query length threshold of 0.9, and a sequence similarity step range of 0, 10, 20, 30, 40, 50, 60, 70, 80, 90, and 95%. (A) The proportion of genomes in which gene families are present. Gene families are considered stable (blue) when they have only a single allele at 98% amino acid identity and diverged (red) when they have >1 allele. (B) The number of unique alleles at each amino acid percentage threshold. A unique allele is characterized as the highest percentage identity threshold at which a unique sub-cluster of isolates from a single gene family was identified by MCL. (C) The number of gene families per isolate ordered alongside the phylogenetic tree. (D) Shared gene presence per isolate ordered alongside the phylogenetic tree. Gene family presence is indicated by a blue block per column. Phylogenetic trees were generated from a core gene alignment from PIRATE and constructed using rapidnj [22].

PIRATE was applied to a dataset of 45 draft genomes of *Prochlorococcus marinus*, a marine cyanobacterium with extremely diverse gene complement, from the NCBI database (Supplementary Table 2) [21]. The pangenome of *P. marinus* was reconstructed, including gene sequence alignment, in 2,976 s (50 min) using 8 threads, an MCL inflation value of 6, and a range of sequence similarity thresholds from 0 to 95% (0, 10, 20, 30, 40, 50, 60, 70, 80, 90, and 95%). This relaxed range of sequence similarity thresholds allowed us to test the lower limits of BLAST/DIAMOND for detecting homology in these data. The pangenome comprised 91,593 loci clustered into 8,325 gene clusters of which 867 (10.41%) were considered core (present in >95% of isolates) (Fig. 5A). There were a large number of genes present at intermediate frequency, most likely due to strong phylogeny structure within the limited sample size, and large numbers of genes private to related lineages. The number of unique alleles per genome increased at percentage identity thresholds of >70%, representing the inter-lineage divergence, and increased sharply at thresholds >94–95%, which is consistent with the sharp intra-species increase in allelic diversity observed in other species in this study (Fig. 5B). The majority of *P. marinus* isolates had a pangenome size of ∼1,800 genes per isolate with the exception of a single lineage that contained ∼2,600 genes (Fig. 5C). Interestingly, the additional genetic complement of this lineage was not composed primarily of genes shared between all isolates; instead it contained a large proportion of rare genes (Fig. 5D). Observation of the number of shared genes alongside the core genome phylogenetic tree of *P. marinus* revealed that each of the deep branching lineages has a complement of approximately equal numbers of lineage core genes (Fig. 5D).

## Conclusion

Here we present PIRATE, a toolbox for pangenomic analysis of bacterial genomes, which provides a framework for exploring gene diversity by defining genes using relaxed sequence similarity thresholds. This pipeline builds upon existing tools using a novel methodology that can be applied to any annotated genomic features. PIRATE identifies and categorizes duplicated and disrupted genes, estimates allelic diversity, scores gene divergence, and contextualizes genes using a pangenome graph. We demonstrate that it compares favourably with other commonly used tools for pangenomic analysis, in both execution time and computational resources, and is fully compatible with software for downstream analysis and visualization. Furthermore, it is scalable to multiprocessor environments and can be applied to large numbers of genomes on modest hardware. Together the enhanced core and accessory genome characterization capability, and the practical implementation advantages, make PIRATE a potentially powerful tool in bacterial genomics - a field in which there is an urgent need for tools that are applicable to increasingly large and complex datasets.

## Availability of Supporting Source Code and Requirements

Project name: PIRATE: A fast and scalable pangenomics toolbox for clustering diverged orthologues in bacteria

Project home page: https://github.com/SionBayliss/PIRATE

Operating system(s): Ubuntu 16.04/18.04, MacOS

Programming language: Perl, R

Other requirements: mcl, mafft, cd-hit, fasttree, ncbi-blast+, bioperl, GNU parallel, diamond

License: GNU GPL v3.0

RRID: SCR_017625

An archival copy of the code, scripts, and other supporting data are also available via the GigaScience database GigaDB [19].

## Additional files

Supplementary Information. Benchmarking analysis and expanded details on the methods used in the PIRATE pipeline.

Supplementary Table 2. Accession numbers for samples used in the benchmarking analysis.

Supplementary Table 3. Parameters used in the analysis of comparisons between PIRATE, Roary and PanX.

## Abbreviations

BLAST: Basic Local Alignment Search Tool; GFF: General Feature Format; HSP: High-Scoring Pair; MAFFT: Multiple Alignment using Fast Fourier Transform; Mb: Megabase Pairs; MCL: Markov Cluster; NCBI: National Center for Biotechnology Information; PIRATE: Pangenome Iterative Refinement and Threshold Evaluation.

## Competing Interests

The authors declare that they have no competing interests.

## Supplementary Material

giz119_GIGA-D-19-00122_Original_Submission

giz119_GIGA-D-19-00122_Revision_1

giz119_GIGA-D-19-00122_Revision_2

giz119_GIGA-D-19-00122_Revision_3

giz119_Response_to_Reviewer_Comments_Original_Submission

giz119_Response_to_Reviewer_Comments_Revision_1

giz119_Response_to_Reviewer_Comments_Revision_2

giz119_Reviewer_1_Report_Original_Submission Andrew Page -- 5/7/2019 Reviewed

giz119_Reviewer_2_Report_Original_Submission Richard Neher -- 5/11/2019 Reviewed

giz119_Reviewer_2_Report_Revision_1 Richard Neher -- 8/7/2019 Reviewed

giz119_Reviewer_3_Report_Original_Submission Jason Sahl -- 5/16/2019 Reviewed

giz119_Reviewer_3_Report_Revision_1 Jason Sahl -- 7/31/2019 Reviewed

giz119_Supplemental_Files

## References

[1] Sheppard SK, Guttman DS, Fitzgerald JR Population genomics of bacterial host adaptation. Nat Rev Genet. 2018;19:549–65. 29973680 10.1038/s41576-018-0032-z

[2] Page AJ, Cummins CA, Hunt M, et al. Roary: rapid large-scale prokaryote pan genome analysis. Bioinformatics. 2015;31:3691–3. 26198102 10.1093/bioinformatics/btv421 PMC4817141

[3] Thorpe HA, Bayliss SC, Sheppard SK, et al. Piggy: a rapid, large-scale pan-genome analysis tool for intergenic regions in bacteria. Gigascience. 2018;7:1–11. 10.1093/gigascience/giy015 PMC5890482 29635296

[4] Sahl JW, Caporaso JG, Rasko DA, et al. The large-scale blast score ratio (LS-BSR) pipeline: a method to rapidly compare genetic content between bacterial genomes. PeerJ. 2014;2:e332. 24749011 10.7717/peerj.332 PMC3976120

[5] Li L, Stoeckert CJ Jr, Roos DS OrthoMCL: identification of ortholog groups for eukaryotic genomes. Genome Res. 2003;13:2178–89. 12952885 10.1101/gr.1224503 PMC403725

[6] Ding W, Baumdicker F, Neher RA panX: pan-genome analysis and exploration. Nucleic Acids Res. 2018;46:e5. 29077859 10.1093/nar/gkx977 PMC5758898

[7] Sheppard SK, Jolley KA, Maiden MCJ A gene-by-gene approach to bacterial population genomics: whole genome MLST of *Campylobacter*. Genes. 2012;3:261–77. 24704917 10.3390/genes3020261 PMC3902793

[8] Méric G, Yahara K, Mageiros L, et al. A reference pan-genome approach to comparative bacterial genomics: identification of novel epidemiological markers in pathogenic *Campylobacter*. PLoS One. 2014;9:e92798. 24676150 10.1371/journal.pone.0092798 PMC3968026

[9] Lees JA, Harris SR, Tonkin-Hill G, et al. Fast and flexible bacterial genomic epidemiology with PopPUNK. Genome Res. 2019;29:304–16. 30679308 10.1101/gr.241455.118 PMC6360808

[10] Denamur E, Matic I. Evolution of mutation rates in bacteria. Mol Microbiol. 2006;60:820–7. 16677295 10.1111/j.1365-2958.2006.05150.x

[11] Li W, Godzik A CD-HIT: a fast program for clustering and comparing large sets of protein or nucleotide sequences. Bioinformatics. 2006;22:1658–9. 16731699 10.1093/bioinformatics/btl158

[12] Buchfink B, Xie C, Huson DH Fast and sensitive protein alignment using DIAMOND. Nat Methods. 2015;12:59–60. 25402007 10.1038/nmeth.3176

[13] Camacho C, Coulouris G, Avagyan V, et al. BLAST+: architecture and applications. BMC Bioinformatics. 2009;10:421. 20003500 10.1186/1471-2105-10-421 PMC2803857

[14] Enright AJ, Van Dongen S, Ouzounis CA . An efficient algorithm for large-scale detection of protein families. Nucleic Acids Res. 2002;30:1575–84. 11917018 10.1093/nar/30.7.1575 PMC101833

[15] Katoh K, Misawa K, Kuma K-I, et al. MAFFT: a novel method for rapid multiple sequence alignment based on fast Fourier transform. Nucleic Acids Res. 2002;30:3059–66. 12136088 10.1093/nar/gkf436 PMC135756

[16] Hadfield J, Croucher NJ, Goater RJ, et al. Phandango: an interactive viewer for bacterial population genomics. Bioinformatics. 2018;34(2):292–3. 10.1093/bioinformatics/btx610 PMC5860215 29028899

[17] Brynildsrud O, Bohlin J, Scheffer L, et al. Rapid scoring of genes in microbial pan-genome-wide association studies with Scoary. Genome Biol. 2016;17:238. 27887642 10.1186/s13059-016-1108-8 PMC5124306

[18] Argimón S, Abudahab K, Goater RJE, et al. Microreact: visualizing and sharing data for genomic epidemiology and phylogeography. Microb Genom. 2016;2:e000093. 28348833 10.1099/mgen.0.000093 PMC5320705

[19] Bayliss SC, Thorpe HA, Coyle NM, et al. Supporting data for “PIRATE: A fast and scalable pangenomics toolbox for clustering diverged orthologues in bacteria.”. GigaScience Database. 2019 10.5524/100645. PMC6785682 31598686

[20] Connor TR, Loman NJ, Thompson S, et al. CLIMB (the Cloud Infrastructure for Microbial Bioinformatics): an online resource for the medical microbiology community. Microb Genom. 2016;2:e000086. 28785418 10.1099/mgen.0.000086 PMC5537631

[21] Pruitt KD, Tatusova T, Maglott DR NCBI reference sequences (RefSeq): a curated non-redundant sequence database of genomes, transcripts and proteins. Nucleic Acids Res. 2007;35:D61–5. 17130148 10.1093/nar/gkl842 PMC1716718

[22] Simonsen M, Mailund T, Pedersen CNS Rapid Neighbour-Joining. Algorithms in Bioinformatics. 2008:113–22.

