# Supplementary material for: PIRATE: A fast and scalable pangenomics toolbox for clustering diverged orthologues in bacteria
Source: Gigascience. 2019 Oct 9;8(10):giz119. doi: 10.1093/gigascience/giz119 (PMC6785682; doi:10.1093/gigascience/giz119)
Supplement: giz119_GIGA-D-19-00122_Revision_1 [file giz119_giga-d-19-00122_revision_1.pdf]

GigaScience

PIRATE: A fast and scalable pangenomics toolbox for clustering diverged orthologues in bacteria

--Manuscript Draft--

**Manuscript Number:**	GIGA-D-19-00122R1
**Full Title:**	PIRATE: A fast and scalable pangenomics toolbox for clustering diverged orthologues in bacteria
**Article Type:**	Technical Note
**Funding Information:**	Biotechnology and Biological Sciences Research Council (BB/M026388/1) — Dr Edward J Feil<br>Medical Research Council (MR/L015080/1) — Dr Samuel K Sheppard
**Abstract:**	Cataloguing the distribution of genes within natural bacterial populations is essential for understanding evolutionary processes and the genetic basis of adaptation. Here we present a pangenomics toolbox, PIRATE (Pangenome Iterative Refinement And Threshold Evaluation), which identifies and classifies orthologous gene families in bacterial pangenomes over a wide range of sequence similarity thresholds. PIRATE builds upon recent scalable software developments to allow for the rapid interrogation of thousands of isolates. PIRATE clusters genes (or other annotated features) over a wide range of amino-acid or nucleotide identity thresholds and uses the clustering information to rapidly classify paralogous gene families into either putative fission/fusion events or gene duplications. Furthermore, PIRATE orders the pangenome using a directed graph, provides a measure of allelic variation and estimates sequence divergence for each gene family. We demonstrate that PIRATE scales linearly with both number of samples and computation resources, allowing for analysis of large genomic datasets, and compares favorably to other popular tools. PIRATE provides a robust framework for analysing bacterial pangenomes, from largely clonal to panmictic species.
**Corresponding Author:**	Sion C Bayliss, Ph.D<br>University of Bath<br>UNITED KINGDOM
**Corresponding Author Secondary Information:**	
**Corresponding Author's Institution:**	University of Bath
**Corresponding Author's Secondary Institution:**	
**First Author:**	Harry A Thorpe
**First Author Secondary Information:**	
**Order of Authors:**	Harry A Thorpe
	Nicola M Coyle
	Samuel K Sheppard
	Edward J Feil
	Sion C Bayliss, Ph.D
**Order of Authors Secondary Information:**	
**Response to Reviewers:**	I would like to thank the editors for considering the manuscript for publication and the reviewers for their time and insightful contributions. I hope that the revisions detailed below contribute to an improved manuscript which will be of broad interest to researchers interested in the field of bacterial genomics.

Powered by Editorial Manager® and ProduXion Manager® from Aries Systems Corporation

Editor Notes:
Comment 1) Please register any new software application in the SciCrunch.org database to receive a RRID (Research Resource Identification Initiative ID) number, and include this in your manuscript (in the "software availability" section). This will facilitate tracking, reproducibility and re-use of your tool.

- “PIRATE is available as a software application in the SciCrunch.org database (RRID SCR_017265)” added to the ‘Software Availability’ section.

Reviewer #1: This is a well written paper describing a new pan-genome method called PIRATE. They compare it to the state of the art and provide many improvements, so it will be of great use to the microbial bioinformatics community. In particular they use Dimond to speed up comparisons, and do a much better job with paralogs and assembly errors compared to Roary (my tool). The software is easy to install via Conda, and it accepts a very commonly used annotation file format (GFF3 files from PROKKA), all things that are often overlooked in papers, so good work.

Comment 1) You should consider adding the GC content of the 3 species under test since there is a nice range.

- Line 152 was amended to “Three bacterial species were selected for comparison, Campylobacter jejuni, Staphylococcus aureus and Escherichia coli, representing both a range of pangenome sizes (small, medium and large respectively) and GC contents (30.4%, 32.7% and 50.6% respectively)(Supplementary Table 2)”.

Comment 2) PopPunk (Lees et al.) has recently come out and would be a relevant citation:

- PopPunk has been added as a citation in the introduction. Additionally Line 252 was changed to “The large increase in the size of the accessory genome content inferred using Roary is primarily due to the post-processing (paralog splitting) of accessory genes and has also been described in previous studies [9].”

Comment 3) Could you add the inflation factor for MCL and how you arrived at it, because it can have a big impact on the end results.

- This was an important parameter for which the inclusion the default parameter was overlooked in the original manuscript. The following text was added to the Methods section, Line 89 - “A default MCL inflation value of 2 was identified as appropriate for intra-species clustering by this study and previous authors . A larger inflation value maybe appropriate for inter-species comparisons and can be modified within the software.”.

Reviewer #2: This manuscript presents the pangenomic analysis pipeline PIRATE, benchmarks it and compares it to other tools roary and panX (I am one of the developers of panX). The tool implements a series of sensible steps to cluster annotated features into orthologous groups.

Comment 1) Benchmarking is a little underwhelming. The tool is marketed as being able to deal with genome collection at many different degrees of divergence and diversity. Testing on just 253 Staph aureus genomes doesn't really do this justice, the results for E coli and Campy are restricted to performance in the supplement. Why not also test on very diverse species (like Plochlorococcus marinus) or entire orders such as Pseudomonadales. The comparisons between tools are also restricted to two numbers (core and accessory genes). More informative comparisons would be between cluster size distribution and some analysis of whether the different tools

actually found the same clusters.

- We thank the reviewer for this comment and have applied PIRATE to two additional example datasets, 48 draft genomes of Prochlorococcus marinus and a collection of 497 complete genomes of Pseudomonas species. For brevity the results of these analyses are included in the ‘Additional Examples’ section of the Supplementary Analysis (Supplementary Figures 7+8) and the presence of these additional examples have been alluded to in the ‘Application to Real Data’ section of the main text at Line 273 - “Additional examples of real data processed using PIRATE have been included in the Supplementary Analysis to highlight application of the tool to large or diverse datasets (Supplementary Figures 7+8). PIRATE was applied to 48 draft genomes of Prochlorococcus marinus, a marine cyanobacteria with extremely diverse gene complement, and a collection of 497 complete genomes of assorted Pseudomonas species, a genus of Gram-negative Gammaproteobacteria with highly variable genome sizes.”.

In addition to this we have performed some additional clustering analysis which has been detailed in the Supplementary Analysis (Section: , Figure 9). The text “An analysis of the clusters produced by the tools indicated that there was broad intersection between metholodogies when considering core genes, but that differences become more pronounced in the intermediate and accessory pangenome (Supplementary Analysis, Supplementary Figure 9).” was added at Line 255 to address the relevant finding in the main text.

Comment 2) Page 1, last paragraph: The issue of overclustering/underclustering should be discussed a little more. In particular, I think the authors should highlight the fact that there is no objective truth to compare against and what is considered a useful clustering output to some extent depends on the downstream analysis.

- Many thanks to the reviewer for this excellent comment, this is a very relevant point which improves the readers’ understanding of the scope of the work. The following lines have been added to the introduction at Line 54 - “The impact of over- and under-clustering is relevant to consider in the context of downstream research applications. Under-clustering (or over-splitting) can create a misleading impression of pangenome diversity and composition when considering how much gene diversity exists in the accessory genome [9]. However, for a study identifying genetic determinants associated with a phenotype, such as antibiotic resistance, core and accessory allelic variation which has been misclassified as additional accessory genes may have little to no impact as the causative genes in question may still be still correctly identified.”

Comment 3) PanX runtimes are quadratic due to all against all comparison. But in the divide and conquer mode, runtimes are linear other than for the tree building step. Using runtimes when running panX with `-dmdc` would be a more appropriate comparison.

- In order to make the methodological comparison more appropriate the benchmarking of PanX was rerun using -dmdc and –subset_size (set to #samples/#threads). The results do not substantially change the comparisons between the tools but the execution time of PanX is significantly reduced. The results have been updated in the relevant panels in Figure 2 and the text in the main manuscript amended:

a) Line 174 added “In order to aid comparison PanX was used with the -dmdc option which allows multithreading of DIAMOND. Without this option the run time of PanX scales quadratically and is inappropriate for larger datasets and comparison to the other tools.”

b) The paragraph following Figure 2 (Line 181) has been slightly amended to more clearly delineate between PIRATE using BLAST or DIAMOND. It now reads:
“The execution time of Roary and PIRATE scaled in an approximately linear manner with increasing number of samples (Figure 2.A). PanX scaled super-linearly, making application to larger datasets potentially problematic. Roary and PIRATE were faster than PanX at all time points without gene-by-gene alignment. The execution time of

Powered by Editorial Manager® and ProduXion Manager® from Aries Systems Corporation

	PIRATE using DIAMOND was comparable to that of Roary without gene-by gene alignment (Figure 2.A, top panel). Roary completed marginally quicker than PIRATE using BLAST without gene-by-gene alignment at all sample sizes. When gene-by-gene alignment was applied both Roary and PIRATE scaled sub-linearly with number of samples, however PIRATE using DIAMOND or BLAST completed substantially faster than either Roary or PanX (Figure 2.A, bottom panel). PIRATE exhibited lower memory usage than the other tools tested, scaling sub-linearly with number of samples (Figure 2.B). In conclusion, PIRATE compared favourably in both execution time and memory usage and these metrics suggest PIRATE can be flexibly applied to large datasets on routinely available hardware"<br><br>Comment 4) On page 4, line 5, you state that panX requires an alignment for paralog splitting. This is correct. But it also requires a tree. This is where the main computational overhead comes from.<br><br>- Line 169 has been amended to "It should be noted that both PIRATE and Roary include post-processing of paralogs in the comparison without alignment or phylogentic tree reconstruction, producing a complete output. PanX does not do this, as alignment, followed by tree building, is a necessary step in paralog identification in this pipeline. "<br><br>Comment 5) Fig 3D is rather unhelpful and difficult to parse in its current form. All relevant parse happen in a tiny fraction of the figure and the 0-line for the upper part has to be guessed.<br><br>- Figure 3D has been amended with rescaled axis in order to provide more space for the relevant information and to provide an easily observed zero value on the x-axis of the top panel.<br><br>Comment 6) After some fiddling, I could get the pipeline to work. See problems I encountered below.<br><br>- I have updated the installation instructions (github README) to specify that the bioconda channels should be added before installing PIRATE in order to clear up any confusion (below):<br><br>conda config --add channels defaults<br>conda config --add channels bioconda<br>conda config --add channels conda-forge<br><br>Reviewer #3: PIRATE represents an interesting method to conduct pan-genomics by comparing the number of clusters at different clustering thresholds. I installed the software easily through CONDA and it seemed to work well on the datasets that I tested.<br><br>Comment 1) Reading the paper, I would have liked to see further delineation between PIRATE and other tools. For example, what are the biological ramifications of large cluster sizes at lower identities? I realize that this paper really discusses the method and not the applications, but some application would be helpful on how different clustering thresholds affect the interpretation.<br><br>We have expanded upon the examples as suggested by the referee (Line 209):<br><br>"PIRATE can quickly be used to identify genes with both highly conserved or divergent sequence similarity or variable copy number. The biological ramifications of these genes will vary between applications. A number of the genes exhibiting high amino acid sequence divergence have been well studied. For example the the core 'accessory regulator' agr locus exhibited a range of sequence identity clustering thresholds; agrA clusters at 91 %, agrB and agrC at 65 % and agrD at 45 % amino acid

Powered by Editorial Manager® and ProduXion Manager® from Aries Systems Corporation

	identity, each with a copy number of 1. We identified that another gene, ArlR, which is known to interact with the agr locus, has a similarly low amino acid similarity of 45 % perhaps implying that the linked genes have undergone similarly patterns of diversifying selection. This example highlights how diversification may lead to over-splitting of genes if only a single sequence identity threshold were used, even if this threshold were applicable to the vast majority of genes in the pangenome. Expansion of families of MGEs or individual genes within the population can also be identified from the outputs. For example, IS256, known to play a role in biofilm formation and resistance to various antimicrobials, is present in 35 genomes, has a conserved amino acid sequence (<2% divergence) but a variable copy number of between 1 to 32 copies within the genomes in which it is present. Using these data is is possible to identify the strains which have an increased dosage of IS256."<br><br><br>Comment 2) I did have some questions about the time to run PIRATE. The manuscript suggests that it is faster than Roary using either blast or diamond. When I run Roary and PIRATE on your set of 100 E. coli genomes using default parameters and 8 processors, I find that Roary finished in 21m46s and PIRATE finished in 1h14m.<br><br>- The run time of PIRATE is faster than roary when alignment (roary: -en, PIRATE: -a) is toggled on. This is because alignment is fully parallelized in PIRATE. PIRATE running on default parameters will not be faster than roary without alignment as PIRATE follows many of the steps in the roary pipeline, plus the addition of multiple MCL thresholds followed by paralog identification and classification (which is more computationally expensive than the paralog splitting of roary due to the use of CDHIT and BLAST on a per cluster basis). To more explicitly address this point Line 184 was modified to "The execution time of PIRATE using DIAMOND was comparable to that of Roary without gene-by gene alignment (Figure 2.A, top panel). Roary completed marginally faster than PIRATE using BLAST without gene-by-gene alignment at all sample sizes." and Line 186 to "When gene-by-gene alignment was applied both Roary and PIRATE scaled sub-linearly with number of samples, however PIRATE completed substantially faster than Roary and PanX (Figure 2.A, bottom panel)."<br><br><br>Comment 3) There may also be some issues scaling with genome diversity. For example, running PIRATE on 61 Orientia tsutsugamushi genomes with default PIRATE parameters, took over 4 hours to complete: "PIRATE completed in 14803s". This makes me worry about the scalability of the algorithm to larger, complex datasets. I think that additional benchmarking on large and complex datasets would help convince me that this method will scale with increasingly large datasets.<br><br>- The time to complete PIRATE scales linearly with sample size. Large numbers of paralogous genes, either real or caused by poor assemblies, will increase run time. For the purpose of the current manuscript all samples have been run on default settings. This may be suboptimal for more diverse datasets. There are various options available to reduce the potential runtime of PIRATE for large or diverse datasets, such as excluding HSPs that are not below a set proportion of the query length, increasing the MCL inflation value to reduce over-clustering (and therefore reduce paralogs) or by using DIAMOND as a faster alternative to BLAST. In order to address these concerns I have run PIRATE on two additional diverse collections of bacterial genomes using some of the options to reduce execution time. These included 497 complete genomes of the genus Pseudomonas that were available from the RefSeq database and a dataset of 48 Prochlorococcus marinus draft genomes, a diverse bacterial species suggested by Reviewer 1. Prochlorococcus marinus was used in preference to Orientia tsutsugamushi due to a larger number of publicly available genomes with a similarly large and diverse accessory genome. PIRATE completed in 2976s (50 mins) for the Prochlorococcus marinus dataset and 188,216s (52.3h) for the larger Pseudomonas dataset. We believe that these run times are consistent with the application of PIRATE to large and/or diverse datasets using accessible hardware. Additional results have been added in the 'Additional Examples' section of the Supplementary Analysis (Supplementary Figures 7+8).
**Additional Information:**	
**Question**	**Response**

Powered by Editorial Manager® and ProduXion Manager® from Aries Systems Corporation

Are you submitting this manuscript to a special series or article collection?	No
**Experimental design and statistics**<br><br>Full details of the experimental design and statistical methods used should be given in the Methods section, as detailed in our Minimum Standards Reporting Checklist. Information essential to interpreting the data presented should be made available in the figure legends.<br><br>Have you included all the information requested in your manuscript?	Yes
**Resources**<br><br>A description of all resources used, including antibodies, cell lines, animals and software tools, with enough information to allow them to be uniquely identified, should be included in the Methods section. Authors are strongly encouraged to cite Research Resource Identifiers (RRIDs) for antibodies, model organisms and tools, where possible.<br><br>Have you included the information requested as detailed in our Minimum Standards Reporting Checklist?	Yes
**Availability of data and materials**<br><br>All datasets and code on which the conclusions of the paper rely must be either included in your submission or deposited in publicly available repositories (where available and ethically appropriate), referencing such data using a unique identifier in the references and in the "Availability of Data and Materials" section of your manuscript.<br><br>Have you have met the above requirement as detailed in our Minimum	Yes

Standards Reporting Checklist?	

Powered by Editorial Manager® and ProduXion Manager® from Aries Systems Corporation

Manuscript
Click here to access/download;Manuscript;Manuscript.pdf
Click here to view linked References

PIRATE: A fast and scalable pangenomics toolbox for clustering diverged orthologues in bacteria

Sion C. Bayliss[1*], Harry A. Thorpe[1], Nicola M. Coyle[1], Samuel K. Sheppard[1] and Edward J. Feil[1]

[1]The Milner Centre for Evolution, Department of Biology and Biochemistry, University of Bath, Bath BA2 7AY

*To whom correspondence should be addressed.

Abstract

Cataloguing the distribution of genes within natural bacterial populations is essential for understanding evolutionary processes and the genetic basis of adaptation. Here we present a pangenomics toolbox, PIRATE (Pangenome Iterative Refinement And Threshold Evaluation), which identifies and classifies orthologous gene families in bacterial pangenomes over a wide range of sequence similarity thresholds. PIRATE builds upon recent scalable software developments to allow for the rapid interrogation of thousands of isolates. PIRATE clusters genes (or other annotated features) over a wide range of amino-acid or nucleotide identity thresholds and uses the clustering information to rapidly classify paralogous gene families into either putative fission/fusion events or gene duplications. Furthermore, PIRATE orders the pangenome using a directed graph, provides a measure of allelic variation and estimates sequence divergence for each gene family. We demonstrate that PIRATE scales linearly with both number of samples and computation resources, allowing for analysis of large genomic datasets, and compares favorably to other popular tools. PIRATE provides a robust framework for analysing bacterial pangenomes, from largely clonal to panmictic species.

Availability: PIRATE is implemented in Perl and is freely available under a GNU GPL 3 open source license from https://github.com/SionBayliss/PIRATE. PIRATE is available as a software application in the SciCrunch.org database (RRID SCR_017265).

Contact: s.bayliss@bath.ac.uk

Keywords: Microbial genomics, pangenomics, next-generation sequencing, bioinformatics.

Supplementary Information: Supplementary data is available online.

Background

For most bacteria the complement of genes for a given species is far greater than the number of genes in any one strain. Comprising core genes shared by all individuals in a species and accessory genes that are variously present or absent, the pangenome represents a pool of genetic variation that underlies the enormous phenotypic variation observed in many bacterial species. Through horizontal gene transfer, bacteria can acquire genes from this pangenome pool that bestow important traits such as virulence or antimicrobial resistance [1].

Over the last decade, advances in whole genome sequencing technologies and bioinformatic analyses have allowed the cataloguing of genes and intergenic regions that make up the pangenomes of many species [2–9].

Current approaches define genes on the basis of strict sequence identity thresholds [2,3,7,8], e-value cutoffs [5,6] and bit score ratios [4]. However, genes accrue variation at different rates under the influence of positive and purifying selection [10]. Therefore, it is difficult to define a single identity threshold beyond which genes cease to belong to the same family. Relaxed thresholds risk over-clustering of related gene families, whilst conservative thresholds risk over-splitting, by misclassifying highly divergent alleles of the same gene into multiple clusters. Over-splitting is likely to be especially problematic in vertically acquired core genes that have undergone strong diversifying selection or horizontally acquired accessory genes from multiple source populations which share a distant common ancestor. The impact of over- and under-clustering is relevant to consider in the context of downstream research applications. Under-clustering (or over-splitting) can create a misleading impression of pangenome diversity and composition when considering how much gene diversity exists in the accessory genome [9]. However, for a study identifying genetic determinants associated with a phenotype, such as antibiotic resistance, core and accessory allelic variation which has been misclassified as additional accessory genes may have little to no impact as the causative genes in question may still be still correctly identified.

In order to address these considerations we have created the Pangenome Iterative Refinement And Threshold Evaluation (PIRATE) toolbox which evaluates and classifies genetic diversity within the pangenome. PIRATE provides the means to create pangenomes from any annotated features (e.g. CDS, tRNA, rRNA) over a user-defined range of amino acid or nucleotide identity thresholds. PIRATE provides measures of sequence divergence and allelic diversity within the sample. PIRATE also categorises paralogs into duplication and/or fission loci, loci disrupted by an insertion, deletion or nonsense mutation. A consistent nomenclature is applied to allow for the user identify gene clusters which are the product of paralog splitting, duplication or fission, providing additional context on both methodological and evolutionary gene provenance. This rapid, scalable method allows for a comprehensive overview of gene content and allelic diversity within the pangenome.

Methods

Pangenome Construction

The PIRATE pipeline has been summarised as a schematic in Figure 1.A. The input is a set of GFF3 files. Feature sequences are filtered and the dataset is reduced by iterative clustering using CD-HIT [2,11]. The longest sequence from each CD-HIT cluster is used as a representative for sequence similarity searching (BLAST/DIAMOND) [12,13]. The normalised bit scores of the resulting all-vs-all comparisons are clustered using MCL after removing hits which fall below a relaxed threshold of percentage identity (default: 50%) [14]. A default MCL inflation value of 2 was identified as appropriate for intra-species clustering by this study and previous authors [2]. A larger inflation value may be appropriate for inter-species comparisons and can be modified as appropriate. The initial clustering at this lower bounds threshold is used to define putative 'gene families' (Figure 1.B). Initial designations may not represent the final outputs as families containing paralogs maybe subsequently split during the paralog splitting step. MCL clustering is repeated over a range of user specified percentage identity thresholds (default 50-95% amino acid identity, increments of 5). Unique MCL clusters at higher thresholds are used to identify 'unique alleles' (Figure 1.B). Loci may be shared between multiple unique alleles (MCL clusters) at different percentage identity thresholds (e.g. Figure 1.B – Family B). PIRATE uses the highest threshold at which a 'unique allele' is observed to define the shared percentage identity in the resulting outputs.

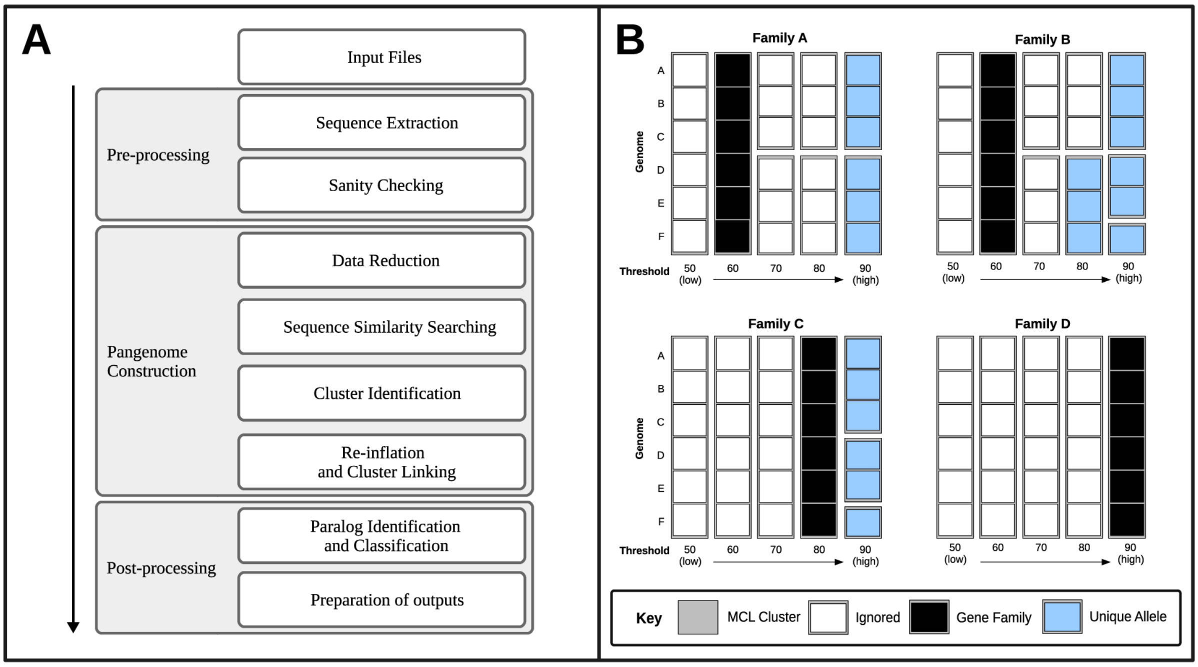

Figure 1. (A) Flow chart denoting a simplified workflow. (B) Example cluster classification. Blocks represent sequences from unique genomes. Grey blocks represent MCL clusters at various percentage identity cut-offs. Black squares indicate a 'gene family' cluster, the lowest %id threshold from the MCL clustering. Blue squares represent 'unique alleles', MCL clusters at higher % identity thresholds with unique combinations of sequences (at the higher threshold at which they are observed together). White squares represent redundant MCL clusters, these are not present in the PIRATE output.

Paralog Classification

Clusters which contain more than one sequence per individual genome are putative paralogs and undergo an additional post-processing step (Supplementary Figure 6). All loci are clustered on the basis of sequence length (98% similar) using CD-HIT. Homology between representative loci is established using all-vs-all BLAST. Loci with no significant overlaps are considered putative fission loci and are compared against a reference sequence (the longest sequence in the gene family) which is considered the most 'complete' version of the gene. All combinations of putative fission loci are

compared to the reference in order to find the combination which gives the most parsimonious coverage of the reference sequence. This combination locus is classified as a ‘fission locus’ that may have formed via gene disruption (e.g. insertion, deletion or nonsense mutation). Any locus which overlaps with all other loci or is not a part of a fission cluster is considered a duplication. The process is iterated until all loci have been classified.

Cluster Splitting

After paralog classification, fission loci are treated as a single locus. Gene families that contain genomes with multiple loci, after accounting for fission loci, potentially represent two or more related gene families that have been over-clustered. In these cases the gene family is checked against the presence of MCL clusters (unique alleles) which contains a single copy of the loci in all constituent genomes (Supplementary Figure 6). These alleles are thereafter considered separate gene families with nomenclature denoting their shared provenance (e.g. g0001_1, g0001_2).

Post-processing

Syntenic connections between gene families in their source genomes are used to create a pangenome graph. Parsimonious paths between gene families contained in the same number of genomes are used to identify co-localised gene families. This information is used to order the resulting tabular pangenome file on syntenic blocks of genes in descending order of number of genomes those blocks were present in. Gene-by-gene alignments are produced using MAFFT in order to generate a core gene alignment [15]. Installing the relevant dependencies in R allows for PIRATE to produce a pdf containing descriptive figures.

A number of supplementary tools are provided to extract, align and subset sequences, and to compare and visualize outputs. In order to facilitate integration with existing pipeline, scripts have been provided to convert the outputs of PIRATE into common formats which allows for them to be used as inputs to software used for downstream analysis, such as the PanX user-interface, SCOARY, Microreact or Phandango [6,16–18]. A full description of the methodology and comparative benchmarks has been provided in the supplementary information (Supplementary Information).

Results and Discussion

Benchmarking and comparison to other tools

The performance of PIRATE was assessed on a range of parameters related to its scalable application to large numbers of bacterial genomes. Three bacterial species were selected for comparison, *Campylobacter jejuni*, *Staphylococcus aureus* and *Escherichia coli*, representing both a range of pangenome sizes (small, medium and large respectively) and GC content (30.4%, 32.7% and 50.6% respectively)(Supplementary Table 2). Memory usage and wall time were found to scale approximately linearly with increasing numbers of isolates and the amount of memory and time per sample was consistent (Supplementary Figures 1+3). PIRATE has been extensively parallelised and the availability of additional cores was found to significantly reduce runtime (Supplementary Figure 2).

A range of tools have been developed for constructing bacterial pangenomes. For comparison, we chose two of the most widely used packages, Roary and PanX [2,6]. These tools have some similarities to PIRATE that facilitate comparison; all three tools share similar clustering workflows (BLAST/DIAMOND, MCL) and require annotated genomes as input. Differences in methodology lie primarily in the post processing of clusters, Roary uses a single percentage identity threshold for MCL clustering and separates paralogs based upon their neighboring genes and PanX splits paralogous genes using an alignment/tree-based method rather than the CDHIT-BLAST approach used by PIRATE. Each of the three tools were applied to subsets of 50, 100, 150, 200 and 250 *Staphylococcus aureus* complete genomes downloaded from the RefSeq database (Supplementary Table 2), for comparisons on the same hardware using 8 cores [19]. It should be noted that both PIRATE and Roary include post-processing of paralogs in the comparison without alignment or phylogenetic tree reconstruction, producing a complete output. PanX does not do this, as alignment, followed by tree building, is a necessary step in paralog identification in this pipeline. Therefore, analyses were run with and without gene-by-gene alignment in order to make unbiased comparisons. Execution time and memory usage per sample were recorded (Figure 2). In order to aid comparison PanX was used with the -dmdc option which allows multithreading of DIAMOND. Without this option the run time of PanX scales quadratically and is inappropriate for larger datasets and comparison to the other tools.

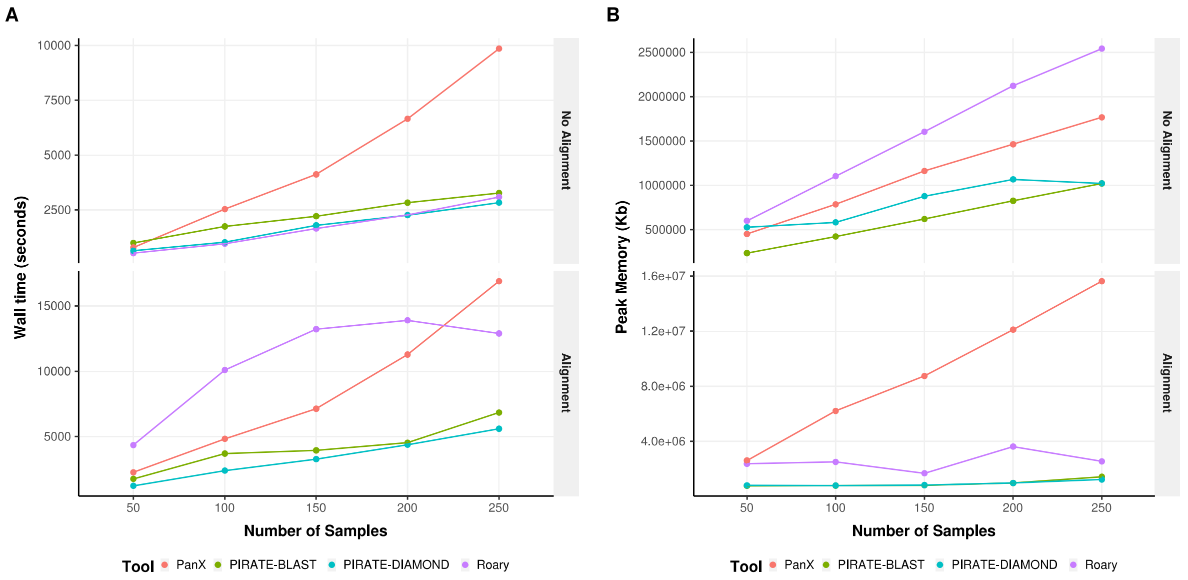

Figure 2. Benchmarking of PIRATE against Roary and PanX. Wall time (seconds) and peak memory usage (Kb) were recorded for each tool run on a dataset of 50, 100, 150, 200 and 250 complete *Staphylococcus aureus* genomes from the RefSeq database with and without gene-by-gene alignment.

The execution time of Roary and PIRATE scaled in an approximately linear manner with increasing number of samples (Figure 2.A). PanX scaled super-linearly, making application to larger datasets potentially problematic. Roary and PIRATE were faster than PanX at all time points without gene-by-gene alignment. The execution time of PIRATE using DIAMOND was comparable to that of Roary without gene-by gene alignment (Figure 2.A, top panel). Roary completed marginally quicker than PIRATE using BLAST without gene-by-gene alignment at all sample sizes. When gene-by-gene alignment was applied both Roary and PIRATE scaled sub-linearly with number of samples, however PIRATE using DIAMOND or BLAST completed substantially faster than either Roary or PanX (Figure 2.A, bottom panel). PIRATE exhibited lower memory usage than the other tools tested, scaling sub-linearly with number of samples (Figure 2.B). In conclusion, PIRATE compared favourably in both execution time and memory usage and these metrics suggest PIRATE can be flexibly applied to large datasets on routinely available hardware.

Application to real data

PIRATE was applied to 253 complete *Staphylococcus aureus* genomes downloaded from the RefSeq database (accessed: 08/11/18) (Supplementary Table 2) [20]. PIRATE was run on default settings over a wide range of amino acid percentage identity thresholds (45, 50, 60, 65, 70, 75, 80, 85, 90, 91-99 in increments of 1%) (Supplementary Table 2). The pangenome of *S. aureus* comprised 4250 gene families of which 2433 (57.25 %) were classified as core (>95% genomes) and 1817 (42.75 %) as accessory (Figure 3.A). Gene families with an average copy number greater than 1.25 loci per genome after paralog classification were excluded from further analysis (178 gene families, 4.18 %) as direct comparison between high copy number or potentially over-clustered families is problematic. Of the remaining 4072 gene families, 740 (18.17 %) clustered at thresholds of less than 95% percentage identity. At these thresholds a significantly different number of 'divergent' gene families were observed (Chi Squared test p-value = < 0.0001) between core and accessory genomes; 21.83 % of accessory genes (383/1754) clustered at less than 95% homology compared to only 15.40 % of core genes (357/2318) (Figure 3.B). A possible explanation for this is that the accessory genes may have been horizontally acquired and therefore may be from diverse genetic backgrounds with different evolutionary histories.

PIRATE can quickly be used to identify genes with both highly conserved or divergent sequence similarity or variable copy number. The biological ramifications of these genes will vary between applications. For example the core 'accessory regulator' *agr* locus exhibited a range of sequence identity clustering thresholds; *agrA* clusters at 91 %, *agrB* and *agrC* at 65 % and *agrD* at 45 % amino acid identity, each with a copy number of 1. We identified that another gene, ArlR, which is known to interact with the *agr* locus, has a similarly low amino acid similarity of 45 % perhaps implying that the linked genes have undergone similarly patterns of diversifying selection. This example highlights how diversification may lead to over-splitting of genes if only a single sequence identity threshold were used, even if this threshold were applicable to the vast majority of genes in the pangenome. Expansion of families of MGEs or individual genes within the population can also be identified from the outputs. For example, IS256, known to play a role in biofilm formation and resistance to various antimicrobials, is present in 35 genomes, has a conserved amino acid sequence (<2% divergence) but a variable copy number of between 1 to 32 copies within the genomes in which it is present. Using these data is is possible to identify the strains which have an increased dosage of IS256.

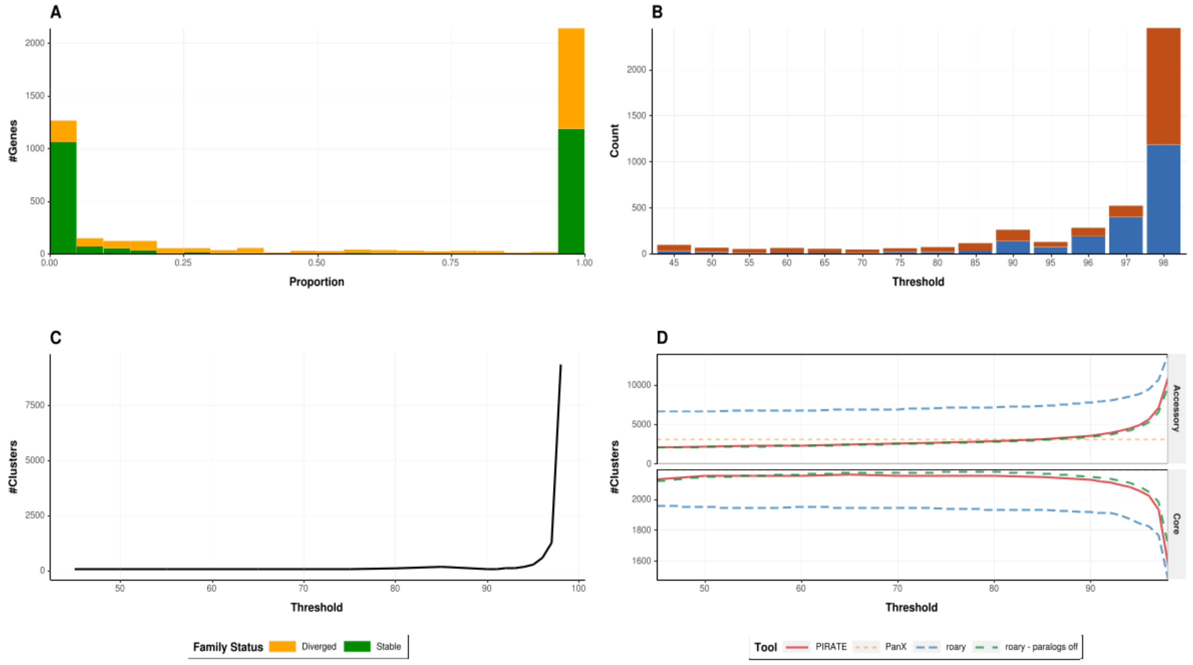

Figure 3. Descriptive figures of the pangenome of 253 complete Staphylococcus aureus genomes inferred using PIRATE. PIRATE was run with default parameters over a range of amino acid identity values (45-98 %). (A) The proportion of genomes in which gene families are found, indicating stable gene families (green) with a single allele at 98% amino acid identity, and diverged with >1 allele (yellow). (B) The minimum amino acid % identity cutoff at which all loci were present per gene family (core = blue, accessory = red). (C) The number of unique alleles at each amino acid percentage threshold. A unique allele is characterised as the highest percentage identity threshold at which a unique sub-cluster of isolates from a single gene family was identified by MCL. (D) Comparison of core and accessory gene/allele estimates for PIRATE (red), PanX (orange), Roary (blue) and Roary with paralog splitting switched off (green). The estimates represent ‘allelic’ variation reported by PIRATE in contrast to ‘gene content’ variation reported by the other tools. PanX provided a single estimate of core and accessory genome content as it has no analogous command to -s in PIRATE or -i in Roary to allow comparison. Core gene families are characterised as being present in greater than 95% of genomes. All tools were run on default parameters. Roary was run over a range of thresholds matching those used for PIRATE with and without paralog splitting (-s).

A steep increase in the number of unique clusters per threshold (allelic diversity) of the sample was observed at thresholds greater than 90% (Figure 3.C). At these thresholds allelic variation will begin to influence the identification of gene families in analogous tools [2,7-8]. In addition to this metric, PIRATE identifies the highest threshold at which all loci in a gene family cluster together. This value can be used to estimate the sequence similarity threshold at which alleles are classified as ‘genes’ by analogous tools (before paralog processing) and therefore allows for evaluation of the influence of this choice on core and accessory genome sizes (Figure 3.D). For comparison, Roary and PanX were applied to the *S. aureus* dataset (default settings). Roary was run at a range of percentage identity thresholds matching those used by PIRATE (-i option) to facilitate comparison. Paralog splitting in Roary was also switched off (-s option) to assess the influence of paralog splitting on the resulting pangenome size estimates. The number of core and accessory genes (<95% isolates) estimated by both tools was compared to those estimated using PIRATE (Figure 3.D). All tools give similar estimates of the number of core genes (PIRATE = 2141, PanX = 2191, Roary (-i 45) = 1959, Roary no paralogs (-i 45) = 2118). However, estimates of the number of accessory genes were divergent (PIRATE = 2190, PanX = 3097, Roary (-i 45) = 6620, Roary no paralogs (-i 45) = 2046). The large increase in the size of the accessory genome content inferred using Roary is primarily due to the post-processing (paralog splitting) of accessory genes and has also been described in previous studies [9]. An analysis of the clusters produced by the tools indicated that there was broad intersection between methodologies when considering core genes, but that differences become more pronounced in the

intermediate and accessory pangenome (Supplementary Analysis, Supplementary Figure 9). The close approximation by PIRATE of accessory content variation in Roary without paralog splitting suggests that PIRATE can be used to provide accurate estimates of pangenome composition for analogous tools before paralog splitting.

For the *S. aureus* collection the estimated number of core genes remains fairly constant at thresholds below 90% and decreases sharply at thresholds greater than 95% (Figure 3.D). This suggests that the majority of the *S. aureus* core genome would be reconstructed by tools that identify genes as clusters of sequences with >10% amino acid sequence similarity. However, the impact of more conservative thresholds on the accessory genome is pronounced. A moderate increase in the number of alleles misidentified as low frequency genes was observed at thresholds <90% followed by a sharp increase at thresholds >90%. This suggests that, even at low homology thresholds, allelic diversity in highly divergent genes inflates the number of clusters incorrectly identified as ‘accessory’ genes when using only a single homology threshold. This effect is likely to be more pronounced in organisms with large accessory genomes due to a higher number of diversified gene families in the accessory genome.

Additional examples of real data processed using PIRATE have been included in the Supplementary Analysis to highlight application of the tool to large or diverse datasets (Supplementary Figures 7+8). PIRATE was applied to 48 draft genomes of *Prochlorococcus marinus*, a marine cyanobacteria with extremely diverse gene complement, and a collection of 497 complete genomes of assorted *Pseudomonas* species, a genus of Gram-negative Gammaproteobacteria with highly variable genome sizes.

Conclusion

Here we present PIRATE, a toolbox for pangenomic analysis of bacterial genomes, which provides a framework for exploring gene diversity by defining genes using relaxed sequence similarity thresholds. This pipeline builds upon existing tools using a novel methodology that can be applied to any annotated genomic feature. PIRATE identifies and categorizes duplicated and disrupted genes, estimates allelic diversity, scores gene divergence and contextualizes genes using a pangenome graph. We demonstrate that it compares favourably with other commonly used tools for pangenomic analysis, in both execution time and computational resources, and is fully compatible with software for downstream analysis and visualisation. Furthermore, it is scalable to multiprocessor environments and can be applied to large numbers of genomes on modest hardware. Together the enhanced core and accessory genome characterisation capability, and the practical implementation advantages, make PIRATE a potentially powerful tool in bacterial genomics - a field in which there is an urgent need for tools that are applicable to increasingly large and complex datasets.

Acknowledgements

We would like to thank everyone who has contributed to the development of PIRATE through testing and feedback.

Funding

This work has been supported by BBSRC/NERC grant BB/M026388/1 awarded to E.F and MRC grant MR/L015080/1 awarded to S.S.

Conflict of Interest: none declared.

Authors Contributions

S.B. developed the software and wrote the manuscript. H.A.T. and N.M.C. contributed to and tested the software. S.K.S. and E.J.F. provided guidance and contributed to the manuscript.

References

1. Sheppard SK, Guttman DS, Fitzgerald JR. Population genomics of bacterial host adaptation. Nat Rev Genet. 2018;19:549–65.

2. Page AJ, Cummins CA, Hunt M, Wong VK, Reuter S, Holden MTG, et al. Roary: rapid large-scale prokaryote pan genome analysis. Bioinformatics. 2015;31:3691–3.

3. Thorpe HA, Bayliss SC, Sheppard SK, Feil EJ. Piggy: a rapid, large-scale pan-genome analysis tool for intergenic regions in bacteria. Gigascience. 2018;7:1–11.

4. Sahl JW, Caporaso JG, Rasko DA, Keim P. The large-scale blast score ratio (LS-BSR) pipeline: a method to rapidly compare genetic content between bacterial genomes. PeerJ. 2014;2:e332.

5. Li L, Stoeckert CJ Jr, Roos DS. OrthoMCL: identification of ortholog groups for eukaryotic genomes. Genome Res. 2003;13:2178–89.

6. Ding W, Baumdicker F, Neher RA. panX: pan-genome analysis and exploration. Nucleic Acids Res. 2018;46:e5.

7. Sheppard SK, Jolley KA, Maiden MCJ. A Gene-By-Gene Approach to Bacterial Population Genomics: Whole Genome MLST of Campylobacter. Genes . 2012;3:261–77.

8. Méric G, Yahara K, Mageiros L, Pascoe B, Maiden MCJ, Jolley KA, et al. A reference pan-genome approach to comparative bacterial genomics: identification of novel epidemiological markers in pathogenic Campylobacter. PLoS One. 2014;9:e92798.

9. Lees JA, Harris SR, Tonkin-Hill G, Gladstone RA, Lo SW, Weiser JN, et al. Fast and flexible bacterial genomic epidemiology with PopPUNK. Genome Res. 2019;29:304–16.

10. Denamur E, Matic I. Evolution of mutation rates in bacteria. Mol Microbiol. 2006;60:820–7.

11. Li W, Godzik A. Cd-hit: a fast program for clustering and comparing large sets of protein or nucleotide sequences. Bioinformatics. 2006;22:1658–9.

12. Buchfink B, Xie C, Huson DH. Fast and sensitive protein alignment using DIAMOND. Nat Methods. 2015;12:59–60.

13. Camacho C, Coulouris G, Avagyan V, Ma N, Papadopoulos J, Bealer K, et al. BLAST+: architecture and applications. BMC Bioinformatics. 2009;10:421.

14. Enright AJ, Van Dongen S, Ouzounis CA. An efficient algorithm for large-scale detection of protein families. Nucleic Acids Res. 2002;30:1575–84.

15. Katoh K, Misawa K, Kuma K-I, Miyata T. MAFFT: a novel method for rapid multiple sequence alignment based on fast Fourier transform. Nucleic Acids Res. 2002;30:3059–66.

16. Hadfield J, Croucher NJ, Goater RJ, Abudahab K, Aanensen DM, Harris SR. Phandango: an interactive viewer for bacterial population genomics. Bioinformatics. 2017;

17. Brynildsrud O, Bohlin J, Scheffer L, Eldholm V. Rapid scoring of genes in microbial pan-genome-wide association studies with Scoary. Genome Biol. 2016;17:238.

18. Argimón S, Abudahab K, Goater RJE, Fedosejev A, Bhai J, Glasner C, et al. Microreact: visualizing and sharing data for genomic epidemiology and phylogeography. Microb Genom. 2016;2:e000093.

19. Connor TR, Loman NJ, Thompson S, Smith A, Southgate J, Poplawski R, et al. CLIMB (the Cloud Infrastructure for Microbial Bioinformatics): an online resource for the medical microbiology community. Microb Genom. 2016;2:e000086.

20. Pruitt KD, Tatusova T, Maglott DR. NCBI reference sequences (RefSeq): a curated non-redundant sequence database of genomes, transcripts and proteins. Nucleic Acids Res. 2007;35:D61–5.

Click here to access/download
Supplementary Material
Supplementary_Analysis.pdf

Supplementary Table 2

Click here to access/download
Supplementary Material
Supplementary_Table_2.tsv

Personal Cover Letter Click here to access/download;Personal Cover;cover_letter.pdf

Dr. Sion C. Bayliss
Milner Centre for Evolution
Department of Biology and Biochemistry
University of Bath
Claverton Down
Bath BA2 7AY
UK

UNIVERSITY OF BATH

29/07/2019
E-mail: s.bayliss@bath.ac.uk
Tel: +44 (0)7838 072372

Dear Editor,

Please find enclosed the manuscript titled ‘PIRATE: A fast and scalable pangenomics toolbox for clustering diverged orthologues in bacteria’, which we would like to be considered for publication as an Technical Note in Gigascience. In this manuscript we describe PIRATE, a software toolbox for pangenomic analysis of bacterial genomes, which provides a framework for exploring the high diversity of genes observed in bacteria. PIRATE uses a novel approach, assessing clusters over a range of sequence similarity thresholds, to define gene orthologues. The software, made freely available for download from Github, identifies and categorizes duplicated and disrupted genes, estimates allelic diversity, scores gene divergence and contextualizes genes using a pangenome graph. PIRATE builds upon existing tools, in both speed and scope, and provides novel and complementary features that can be applied to any annotated genomic feature. In this manuscript we describe the underlying method and demonstrate the utility using a reference collection of *Staphylococcus aureus* genomes, highlighting how the identification of divergent core genes leads to a more conservative estimate of pangenome size. We additionally apply PIRATE to other large and diverse datasets for the purposes of both benchmarking and in order to illustrate the potential applications of the software. Given the rapid technological advances in sequencing technology and the ever expanding number of genomes available from diverse species, PIRATE represents a timely application that will be of broad interest to researchers interested in the field of bacterial genomics.

Yours sincerely,
Dr Sion C. Bayliss, on behalf of all co-authors.
