# Supplementary material for: PIRATE: A fast and scalable pangenomics toolbox for clustering diverged orthologues in bacteria
Source: Gigascience. 2019 Oct 9;8(10):giz119. doi: 10.1093/gigascience/giz119 (PMC6785682; doi:10.1093/gigascience/giz119)
Supplement: giz119_GIGA-D-19-00122_Revision_3 [file giz119_giga-d-19-00122_revision_3.pdf]

GigaScience

PIRATE: A fast and scalable pangenomics toolbox for clustering diverged orthologues in bacteria

--Manuscript Draft--

**Manuscript Number:**	GIGA-D-19-00122R3
**Full Title:**	PIRATE: A fast and scalable pangenomics toolbox for clustering diverged orthologues in bacteria
**Article Type:**	Technical Note
**Funding Information:**	Biotechnology and Biological Sciences Research Council (BB/M026388/1) — Dr Edward J Feil<br>Medical Research Council (MR/L015080/1) — Dr Samuel K Sheppard
**Abstract:**	Cataloguing the distribution of genes within natural bacterial populations is essential for understanding evolutionary processes and the genetic basis of adaptation. Here we present a pangenomics toolbox, PIRATE (Pangenome Iterative Refinement And Threshold Evaluation), which identifies and classifies orthologous gene families in bacterial pangenomes over a wide range of sequence similarity thresholds. PIRATE builds upon recent scalable software developments to allow for the rapid interrogation of thousands of isolates. PIRATE clusters genes (or other annotated features) over a wide range of amino-acid or nucleotide identity thresholds and uses the clustering information to rapidly identify paralogous gene families and putative fission/fusion events. Furthermore, PIRATE orders the pangenome using a directed graph, provides a measure of allelic variation and estimates sequence divergence for each gene family. We demonstrate that PIRATE scales linearly with both number of samples and computation resources, allowing for analysis of large genomic datasets, and compares favorably to other popular tools. PIRATE provides a robust framework for analysing bacterial pangenomes, from largely clonal to panmictic species.
**Corresponding Author:**	Sion C Bayliss, Ph.D<br>University of Bath<br>UNITED KINGDOM
**Corresponding Author Secondary Information:**	
**Corresponding Author's Institution:**	University of Bath
**Corresponding Author's Secondary Institution:**	
**First Author:**	Sion C Bayliss, Ph.D
**First Author Secondary Information:**	
**Order of Authors:**	Sion C Bayliss, Ph.D<br>Harry A Thorpe<br>Nicola M Coyle<br>Samuel K Sheppard<br>Edward J Feil
**Order of Authors Secondary Information:**	
**Response to Reviewers:**	The manuscript has been attached as a .docx formatted file. PDF also attached.<br><br>Editor Notes:<br><br>1) Please add the GigaDB DOI link to your reference [19] in the bibliography (http://dx.doi.org/10.5524/100645):<br>Added to reference.

Powered by Editorial Manager® and ProduXion Manager® from Aries Systems Corporation

	2) Please add a sentence at the end of your "software availability" section along the lines of: "An archival copy of the code, scripts and other supporting data are also available via the GigaScience database GigaDB [19]."<br>Added in text.
**Additional Information:**	
**Question**	**Response**
Are you submitting this manuscript to a special series or article collection?	No
**Experimental design and statistics**<br><br>Full details of the experimental design and statistical methods used should be given in the Methods section, as detailed in our Minimum Standards Reporting Checklist. Information essential to interpreting the data presented should be made available in the figure legends.<br><br>Have you included all the information requested in your manuscript?	Yes
**Resources**<br><br>A description of all resources used, including antibodies, cell lines, animals and software tools, with enough information to allow them to be uniquely identified, should be included in the Methods section. Authors are strongly encouraged to cite Research Resource Identifiers (RRIDs) for antibodies, model organisms and tools, where possible.<br><br>Have you included the information requested as detailed in our Minimum Standards Reporting Checklist?	Yes
**Availability of data and materials**<br><br>All datasets and code on which the conclusions of the paper rely must be either included in your submission or deposited in publicly available repositories (where available and ethically appropriate), referencing such data using	Yes

a unique identifier in the references and in the “Availability of Data and Materials” section of your manuscript.<br><br>Have you have met the above requirement as detailed in our Minimum Standards Reporting Checklist?	

Manuscript
Click here to access/download;Manuscript;Manuscript (1).pdf
Click here to view linked References

PIRATE: A fast and scalable pangenomics toolbox for clustering diverged orthologues in bacteria

Sion C. Bayliss[1]*, Harry A. Thorpe[1], Nicola M. Coyle[1], Samuel K. Sheppard[1] and Edward J. Feil[1]

[1]The Milner Centre for Evolution, Department of Biology and Biochemistry, University of Bath, Bath BA2 7AY

*To whom correspondence should be addressed.

Abstract

Cataloguing the distribution of genes within natural bacterial populations is essential for understanding evolutionary processes and the genetic basis of adaptation. Here we present a pangenomics toolbox, PIRATE (Pangenome Iterative Refinement And Threshold Evaluation), which identifies and classifies orthologous gene families in bacterial pangenomes over a wide range of sequence similarity thresholds. PIRATE builds upon recent scalable software developments to allow for the rapid interrogation of thousands of isolates. PIRATE clusters genes (or other annotated features) over a wide range of amino-acid or nucleotide identity thresholds and uses the clustering information to rapidly identify paralogous gene families and putative fission/fusion events. Furthermore, PIRATE orders the pangenome using a directed graph, provides a measure of allelic variation and estimates sequence divergence for each gene family. We demonstrate that PIRATE scales linearly with both number of samples and computation resources, allowing for analysis of large genomic datasets, and compares favorably to other popular tools. PIRATE provides a robust framework for analysing bacterial pangenomes, from largely clonal to panmictic species.

Availability: PIRATE is implemented in Perl and is freely available under a GNU GPL 3 open source license from https://github.com/SionBayliss/PIRATE. PIRATE is available as a software application in the SciCrunch.org database (RRID SCR_017265).

Contact: s.bayliss@bath.ac.uk

Keywords: Microbial genomics, pangenomics, next-generation sequencing, bioinformatics.

Supplementary Information: Supplementary data is available online.

Background

For most bacteria the complement of genes for a given species is far greater than the number of genes in any one strain. Comprising core genes shared by all individuals in a species and accessory genes that are variously present or absent, the pangenome represents a pool of genetic variation that underlies the enormous phenotypic variation observed in many bacterial species. Through horizontal gene transfer, bacteria can acquire genes from this pangenome pool that bestow important traits such as virulence or antimicrobial resistance [1].

Over the last decade, advances in whole genome sequencing technologies and bioinformatic analyses have allowed the cataloguing of genes and intergenic regions that make up the pangenomes of many species [2–9].

Current approaches define genes on the basis of strict sequence identity thresholds [2,3,7,8], e-value cutoffs [5,6] and bit score ratios [4]. However, genes accrue variation at different rates under the influence of positive and purifying selection [10]. Therefore, it is difficult to define a single identity threshold beyond which genes cease to belong to the same family. Relaxed thresholds risk over-clustering of related gene families, whilst conservative thresholds risk over-splitting, by misclassifying highly divergent alleles of the same gene into multiple clusters. Over-splitting is likely to be especially problematic in vertically acquired core genes that have undergone strong diversifying selection or horizontally acquired accessory genes from multiple source populations which share a distant common ancestor. The impact of over- and under-clustering is relevant to consider in the context of downstream research applications. Under-clustering (or over-splitting) can create a misleading impression of pangenome diversity and composition when considering how much gene diversity exists in the accessory genome [9]. However, for a study identifying genetic determinants associated with a phenotype, such as antibiotic resistance, core and accessory allelic variation which has been misclassified as additional accessory genes may have little to no impact as the causative genes in question may still be correctly identified.

In order to address these considerations we have created the Pangenome Iterative Refinement And Threshold Evaluation (PIRATE) toolbox which evaluates and classifies genetic diversity within the pangenome. PIRATE provides the means to create pangenomes from any annotated features (e.g. CDS, tRNA, rRNA) over a user-defined range of amino acid or nucleotide identity thresholds. PIRATE provides measures of sequence divergence and allelic diversity within the sample. PIRATE also categorises paralogs into duplication and/or fission loci, loci disrupted by an insertion, deletion or nonsense mutation. A consistent nomenclature is applied to allow for the user to identify gene clusters which are the product of duplication or fission events, providing additional context on both methodological and evolutionary gene provenance. This rapid, scalable method allows for a comprehensive overview of gene content and allelic diversity within the pangenome.

Methods

Pangenome Construction

The PIRATE pipeline has been summarised as a schematic in Figure 1.A. The input is a set of GFF3 files. Feature sequences are filtered and the dataset is reduced by iterative clustering using CD-HIT [2,11]. The longest sequence from each CD-HIT cluster is used as a representative for sequence similarity searching (BLAST/DIAMOND) [12,13]. The normalised bit scores of the resulting all-vs-all comparisons are clustered using MCL after removing hits which fall below a relaxed threshold of percentage identity (default: 50%) [14]. A default MCL inflation value of 2 was identified as appropriate for intra-species clustering by this study and previous authors [2]. A larger inflation value may be appropriate for inter-species comparisons and can be modified as appropriate. The initial clustering at this lower bounds threshold is used to define putative 'gene families' (Figure 1.B). Initial designations may not represent the final outputs as families containing paralogs maybe subsequently split during the paralog splitting step. MCL clustering is repeated over a range of user specified percentage identity thresholds (default 50-95% amino acid identity, increments of 5). Unique MCL clusters at higher thresholds are used to identify 'unique alleles' (Figure 1.B). Loci may be shared between multiple unique alleles (MCL clusters) at different percentage identity thresholds (e.g. Figure 1.B – Family B). PIRATE uses the highest threshold at which a 'unique allele' is observed to define the shared percentage identity in the resulting outputs.

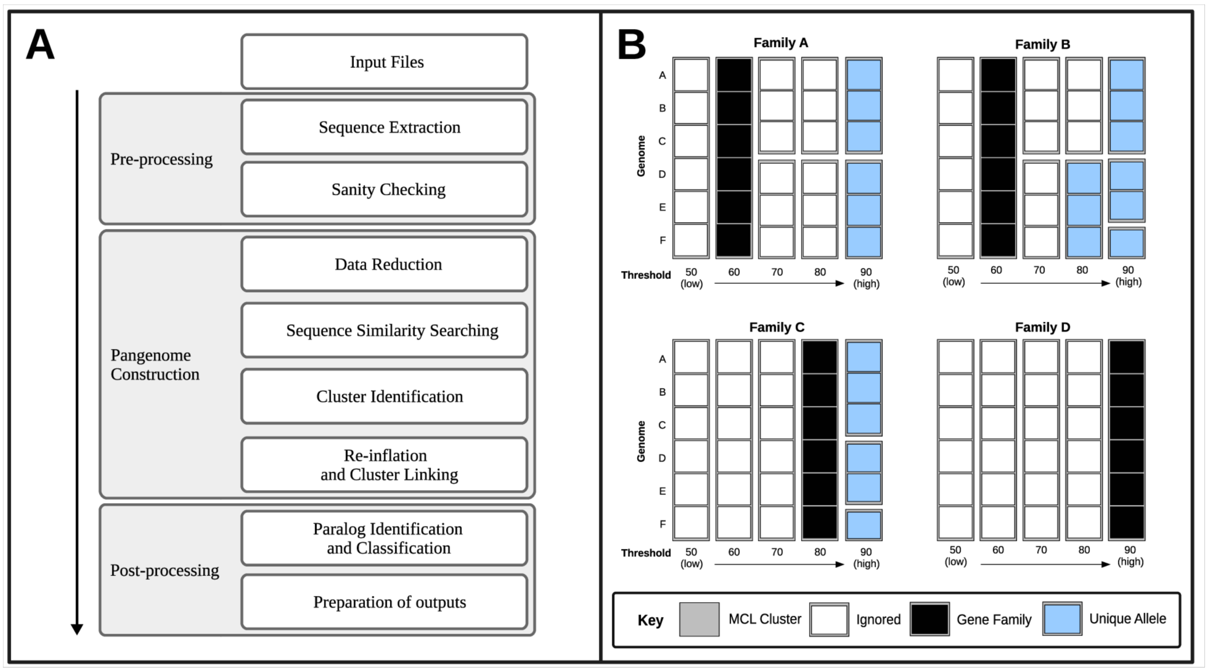

Figure 1. (A) Flow chart denoting a simplified workflow. (B) Example cluster classification. Blocks represent sequences from unique genomes. Grey blocks represent MCL clusters at various percentage identity cut-offs. Black squares indicate a 'gene family' cluster, the lowest %id threshold from the MCL clustering. Blue squares represent 'unique alleles', MCL clusters at higher % identity thresholds with unique combinations of sequences (at the higher threshold at which they are observed together). White squares represent redundant MCL clusters, these are not present in the PIRATE output.

Paralog Classification

Clusters which contain more than one sequence per individual genome are putative paralogs and undergo an additional post-processing step (Supplementary Figure 6). All loci are clustered on the basis of sequence length (98% similar) using CD-HIT. Homology between representative loci is established using all-vs-all BLAST. Loci with no significant overlaps are considered putative fission loci and are compared against a reference sequence (the longest sequence in the gene family) which is considered the most ‘complete’ version of the gene. All combinations of putative fission loci are compared to the reference in order to find the combination which gives the most parsimonious coverage of the reference sequence. This combination locus is classified as a ‘fission locus’ that may have formed via gene disruption (e.g. insertion, deletion or nonsense mutation). Any locus which overlaps with all other loci or is not a part of a fission cluster is considered a duplication. The process is iterated until all loci have been classified.

Cluster Splitting

After paralog classification, fission loci are treated as a single locus. Gene families that contain genomes with multiple loci, after accounting for fission loci, potentially represent two or more related gene families that have been over-clustered. In these cases the gene family is checked against the presence of MCL clusters (unique alleles) which contains a single copy of the loci in all constituent genomes (Supplementary Figure 6). These alleles are thereafter considered separate gene families with nomenclature denoting their shared provenance (e.g. g0001_1, g0001_2).

Post-processing

Syntenic connections between gene families in their source genomes are used to create a pangenome graph. Parsimonious paths between gene families contained in the same number of genomes are used to identify co-localised gene families. This information is used to order the resulting tabular pangenome file on syntenic blocks of genes in descending order of number of genomes those blocks were present in. Gene-by-gene alignments are produced using MAFFT in order to generate a core gene alignment [15]. Installing the relevant dependencies in R allows for PIRATE to produce a pdf containing descriptive figures.

A number of supplementary tools are provided to extract, align and subset sequences, and to compare and visualize outputs. In order to facilitate integration with existing pipeline, scripts have been provided to convert the outputs of PIRATE into common formats which allows for them to be used as inputs to software used for downstream analysis, such as the PanX user-interface, SCOARY, Microreact or Phandango [6,16–18]. A full description of the methodology and comparative benchmarks has been provided in the supplementary information (Supplementary Information).

Results and Discussion

Benchmarking and comparison to other tools

The performance of PIRATE was assessed on a range of parameters related to its scalable application to large numbers of bacterial genomes. Three bacterial species were selected for comparison, *Campylobacter jejuni*, *Staphylococcus aureus* and *Escherichia coli*, representing both a range of pangenome sizes (small, medium and large respectively) and GC content (30.4%, 32.7% and 50.6% respectively)(Supplementary Table 2). The scripts used to perform these analyses are available from the GigaDB repository associated with the publication [19]. The settings used for each tool have been detailed in Supplementary Table 3. Memory usage and wall time were found to scale approximately linearly with increasing numbers of isolates and the amount of memory and time per sample was consistent (Supplementary Figures 1+3). PIRATE has been extensively parallelised and the availability of additional cores was found to significantly reduce runtime (Supplementary Figure 2).

A range of tools have been developed for constructing bacterial pangenomes. For comparison, we chose two of the most widely used packages, Roary and PanX [2,6]. These tools have some similarities to PIRATE that facilitate comparison; all three tools share similar clustering workflows (BLAST/DIAMOND, MCL) and require annotated genomes as input. Differences in methodology lie primarily in the post processing of clusters, Roary uses a single percentage identity threshold for MCL clustering and separates paralogs based upon their neighboring genes and PanX splits paralogous genes using an alignment/tree-based method rather than the CDHIT-BLAST approach used by PIRATE. Each of the three tools were applied to subsets of 50, 100, 150, 200 and 250 *Staphylococcus aureus* complete genomes downloaded from the RefSeq database (Supplementary Table 2), for comparisons on the same hardware using 8 cores [20]. It should be noted that both PIRATE and Roary include post-processing of paralogs in the comparison without alignment or phylogenetic tree reconstruction, producing a complete output. PanX does not do this, as alignment, followed by tree building, is a necessary step in paralog identification in this pipeline. Therefore, analyses were run with and without gene-by-gene alignment in order to make unbiased comparisons. Execution time and memory usage per sample were recorded (Figure 2). In order to aid comparison PanX was used with the -dmdc flag which batches input genomes, clusters per batch and subsequently merges the batches. Without this option the run time of PanX scales quadratically and is inappropriate for larger datasets and comparison to the other tools.

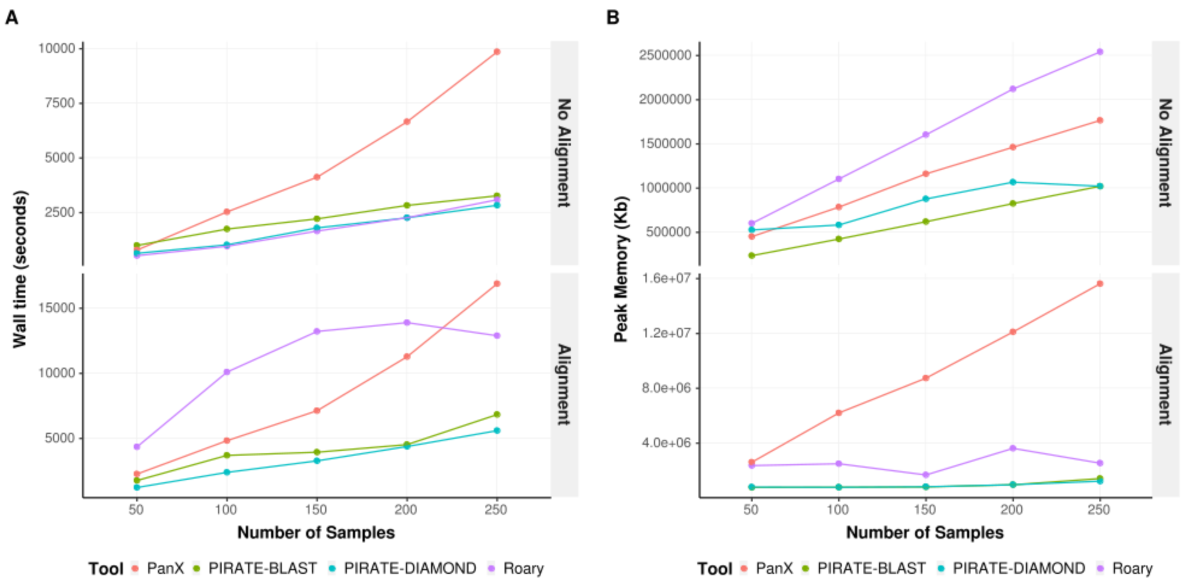

Figure 2. Benchmarking of PIRATE against Roary and PanX. Wall time (seconds) and peak memory usage (Kb) were recorded for each tool run on a dataset of 50, 100, 150, 200 and 250 complete *Staphylococcus aureus* genomes from the RefSeq database with and without gene-by-gene alignment.

The execution time of Roary and PIRATE scaled in an approximately linear manner with increasing number of samples (Figure 2.A). Roary and PIRATE were faster than PanX at all time points without gene-by-gene alignment. The execution time of PIRATE using DIAMOND was comparable to that of Roary without gene-by gene alignment (Figure 2.A, top panel). Roary completed marginally quicker than PIRATE using BLAST without gene-by-gene alignment at all sample sizes. When gene-by-gene alignment was applied both Roary and PIRATE scaled sub-linearly with number of samples, however PIRATE using DIAMOND or BLAST completed substantially faster than either Roary or PanX (Figure 2.A, bottom panel). PIRATE exhibited lower memory usage than the other tools tested, scaling sub-linearly with number of samples (Figure 2.B). In conclusion, PIRATE compared favourably in both execution time and memory usage and these metrics suggest PIRATE can be flexibly applied to large datasets on routinely available hardware.

Application to real datasets

PIRATE has been applied to three real datasets; *Staphylococcus aureus*, *Prochlorococcus marinus* and *Pseudomonas*. *S. aureus*, a gram-positive human commensal and opportunistic pathogen, was used as a benchmarking dataset for comparison to other tools. Additionally, PIRATE was applied to a further two datasets to highlight its application to large or diverse pangenomes. PIRATE was applied to 45 draft genomes of *P. marinus*, a marine cyanobacteria with extremely diverse gene complement, and a collection of 497 complete genomes of assorted *Pseudomonas* species, a genus of Gram-negative *Gammaproteobacteria* which have highly variably sized genomes.

Staphylococcus aureus

PIRATE was applied to 253 complete *Staphylococcus aureus* genomes downloaded from the RefSeq database (accessed: 08/11/18) (Supplementary Table 2) [21]. PIRATE was run on default settings over a wide range of amino acid percentage identity thresholds (45, 50, 60, 65, 70, 75, 80, 85, 90, 91-99 in increments of 1%) (Supplementary Table 2). The pangenome of *S. aureus* comprised 4250 gene

families of which 2433 (57.25 %) were classified as core (>95% genomes) and 1817 (42.75 %) as accessory (Figure 3.A). Gene families with an average copy number greater than 1.25 loci per genome after paralog classification were excluded from further analysis (178 gene families, 4.18 %) as direct comparison between high copy number or potentially over-clustered families is problematic. Of the remaining 4072 gene families, 740 (18.17 %) clustered at thresholds of less than 95% percentage identity. At these thresholds a significantly different number of ‘divergent’ gene families were observed (Chi Squared test p-value = < 0.0001) between core and accessory genomes; 21.83 % of accessory genes (383/1754) clustered at less than 95% homology compared to only 15.40 % of core genes (357/2318) (Figure 3.B). A possible explanation for this is that the accessory genes may have been horizontally acquired and therefore may be from diverse genetic backgrounds with different evolutionary histories.

PIRATE can quickly be used to identify genes with both highly conserved or divergent sequence similarity or variable copy number. The biological ramifications of these genes will vary between applications. For example the core ‘accessory regulator’ *agr* locus exhibited a range of sequence identity clustering thresholds; *agrA* clusters at 91 %, *agrB* and *agrC* at 65 % and *agrD* at 45 % amino acid identity, each with a copy number of 1. We identified that another gene, *arlR*, which is known to interact with the *agr* locus, has a similarly low amino acid similarity of 45 % perhaps implying that the linked genes have undergone similar patterns of diversifying selection. This example highlights how diversification may lead to over-splitting of genes if only a single sequence identity threshold were used, even if this threshold were applicable to the vast majority of genes in the pangenome. Expansion of families of MGEs or individual genes within the population can also be identified from the outputs. For example, IS256, known to play a role in biofilm formation and resistance to various antimicrobials, is present in 35 genomes, has a conserved amino acid sequence (<2% divergence) but a variable copy number of between 1 to 32 copies within the genomes in which it is present. Using these data is is possible to identify the strains which have an increased dosage of IS256.

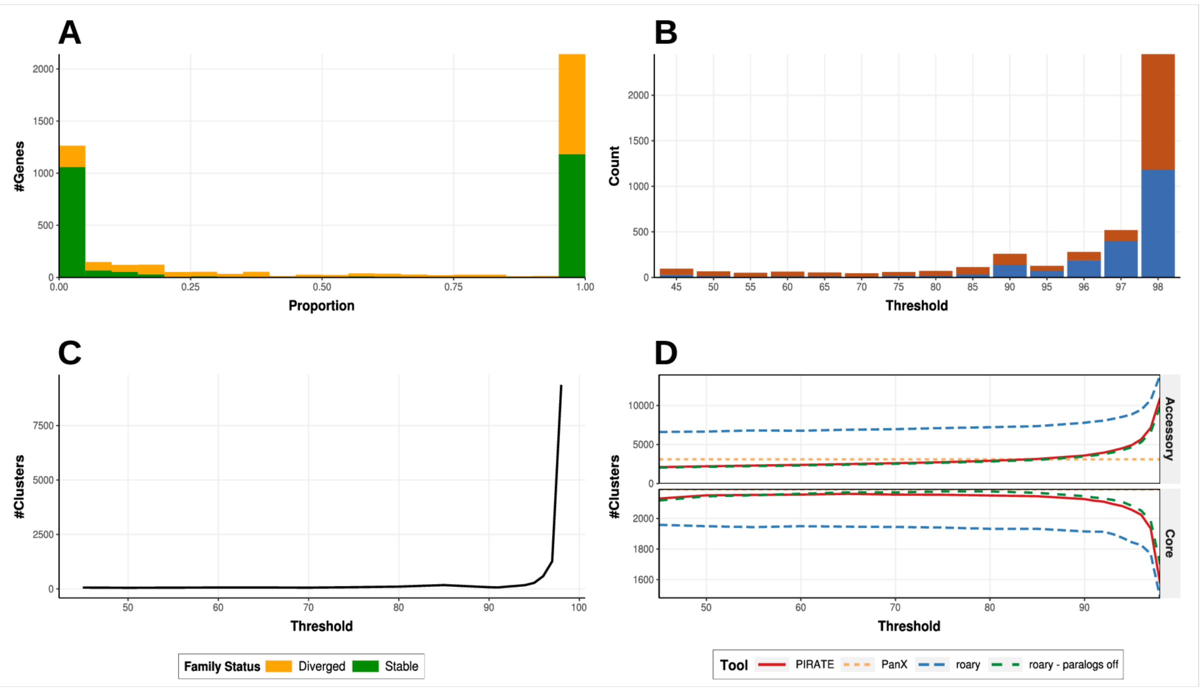

Figure 3. Descriptive figures of the pangenome of 253 complete Staphylococcus aureus genomes inferred using PIRATE. PIRATE was run with default parameters over a range of amino acid identity values (45-98 %). (A) The proportion of genomes in which gene families are found, indicating stable gene families (green) with a

single allele at 98% amino acid identity, and diverged with >1 allele (yellow). (B) The minimum amino acid % identity cutoff at which all loci were present per gene family (core = blue, accessory = red). (C) The number of unique alleles at each amino acid percentage threshold. A unique allele is characterised as the highest percentage identity threshold at which a unique sub-cluster of isolates from a single gene family was identified by MCL. (D) Comparison of core and accessory gene/allele estimates for PIRATE (red), PanX (orange), Roary (blue) and Roary with paralog splitting switched off (green). The estimates represent 'allelic' variation reported by PIRATE in contrast to 'gene content' variation reported by the other tools. PanX provided a single estimate of core and accessory genome content as it has no analogous command to -s in PIRATE or -i in Roary to allow comparison. Core gene families are characterised as being present in greater than 95% of genomes. All tools were run on default parameters. Roary was run over a range of thresholds matching those used for PIRATE with and without paralog splitting (-s).

A steep increase in the number of unique clusters per threshold (allelic diversity) of the sample was observed at thresholds greater than 90% (Figure 3.C). At these thresholds allelic variation will begin to influence the identification of gene families in analogous tools [2,7-8]. In addition to this metric, PIRATE identifies the highest threshold at which all loci in a gene family cluster together. This value can be used to estimate the sequence similarity threshold at which alleles are classified as 'genes' by analogous tools (before paralog processing) and therefore allows for evaluation of the influence of this choice on core and accessory genome sizes (Figure 3.D). For comparison, Roary and PanX were applied to the *S. aureus* dataset (default settings). Roary was run at a range of percentage identity thresholds matching those used by PIRATE (-i option) to facilitate comparison. Paralog splitting in Roary was also switched off (-s option) to assess the influence of paralog splitting on the resulting pangenome size estimates. The number of core and accessory genes (<95% isolates) estimated by both tools was compared to those estimated using PIRATE (Figure 3.D). All tools give similar estimates of the number of core genes (PIRATE = 2141, PanX = 2191, Roary (-i 45) = 1959, Roary no paralogs (-i 45) = 2118). However, estimates of the number of accessory genes were divergent (PIRATE = 2190, PanX = 3097, Roary (-i 45) = 6620, Roary no paralogs (-i 45) = 2046).

For the *S. aureus* collection the estimated number of core genes remains fairly constant at thresholds below 90% and decreases sharply at thresholds greater than 95% (Figure 3.D). This suggests that the majority of the *S. aureus* core genome would be reconstructed by tools that identify genes as clusters of sequences with >10% amino acid sequence similarity. However, the impact of more conservative thresholds on the accessory genome is pronounced. A moderate increase in the number of alleles misidentified as low frequency genes was observed at thresholds <90% followed by a sharp increase at thresholds >90%. This suggests that, even at low identity thresholds, allelic diversity in highly divergent genes inflates the number of clusters incorrectly identified as 'accessory' genes when using only a single homology threshold. This effect is likely to be more pronounced in organisms with large accessory genomes due to a higher number of diversified gene families in the accessory genome.

The outputs from the three tools were compared to identify the differences in the gene clusters that they produced. Loci not present in all outputs, due to tool-specific input sequence filters, were removed. PIRATE produced 4,247 clusters, PanX 5,193 and Roary 10,454 clusters. The clusters were compared in a pairwise manner between tools and the number of matching clusters were identified (Supplementary Figure 7). Clusters were considered matching when they contained the same loci and were +/-5% the size (number of loci) of the query cluster. The relaxed cluster size threshold (+/-5%) was applied to allowed for minor discrepancies between the clusterings that were unlikely to significantly impact on the interpretation of results. The majority of clusters matched between PIRATE and PanX (PanX:PIRATE = 3515/5193 [67.69 %], PIRATE-PanX = 3456/4247 [81.38 %]). Many of mismatches occurred in the accessory or intermediate pangenome. The greater number of

PIRATE clusters identified in the PanX output was likely due to the less aggressive paralog splitting algorithm and co-clustering of truncated genes (fission/fusion genes) used by PIRATE. The majority (~70%) of PIRATE and PanX clusters were found in the output of Roary (PanX:Roary = 3736/5193 [71.94 %], PIRATE:Roary = 2979/4247 [70.14 %]), suggesting that a large proportion of core genes were found by all tools. The smallest number of matching clusters (~25 %) were between Roary and the clusters identified by the other tools (Roary:PanX = 3029/10454 [28.97 %], Roary:PIRATE=2419/10454 [23.14 %]) and most of these mismatches were observed in accessory clusters. We would suggest that this is due to the aggressive splitting of paralogous genes in Roary, the implications of which have been documented by previous authors [9].

These results suggest that there was a large intersection in the core gene clusters and, to a lesser extent, accessory clusters, of the three tools studied. However, the tools varied in the identification of shared clusters in the intermediate and accessory pangenomes. This difference was more pronounced in accessory genes identified by Roary than between PIRATE and PanX. The vast majority of the differences in clustering between tools in most likely due to the different paralog splitting methodologies employed. Other variations in methodology, such as the 'divide-and-conquer' strategy employed by PanX or the co-clustering of fission/fusion genes by PIRATE, may also contribute to this variation to a lesser extent. The close approximation by PIRATE of accessory content variation in Roary without paralog splitting suggests that PIRATE can be used to provide accurate estimates of pangenome composition for analogous tools before paralog splitting.

Pseudomonas Species

PIRATE was applied to a dataset of 496 complete genomes of assorted, uncharacterized *Pseudomonas* species from the NCBI database (Supplementary Table 2)[21]. The pangenome of the *Pseudomonas* collection was reconstructed, including gene-by-gene sequence alignment, in 188,216s (52.3h) using 12 threads, an MCL inflation value of 6 and a HSP query length threshold of 0.9. The pangenome comprised of 2,858,820 loci clustered into 102,425 gene clusters of which 1841 (1.8 %) were considered core (present in >95% of isolates) (Figure 4.A). An increase in the frequency of genes present in ~40% of the isolates corresponded to 'lineage core' genes from an overrepresented lineage (Figure 4.C, dotted blue box). The number of unique alleles per genome increased at percentage identities thresholds >70 %, most likely representing inter-species/lineage divergence, and increased sharply at thresholds >94-95% (Figure 4.B). This rise was consistent with the sharp increase of intra-species allelic diversity observed in other datasets investigated within this study (Figure 4.B). *Pseudomonas* had an extremely variable genome size (4.7-11 Mb) which was reflected in the number of genes present per isolate (Figure 4.C). There was an observable relationship between genetic relatedness and number of genes per isolate with considerable within-lineage variation. This is most clearly observable in the most numerous lineage present in the collection (Figure 4.C, dotted blue box) which contained between 5000-7000 genes per isolate. Whilst there were a large number of 'lineage core' genes present in *Pseudomonas* species, there were also a number of promiscuous genes intermittently present or absent across all *Pseudomonas* genomes analysed (Figure 4.D).

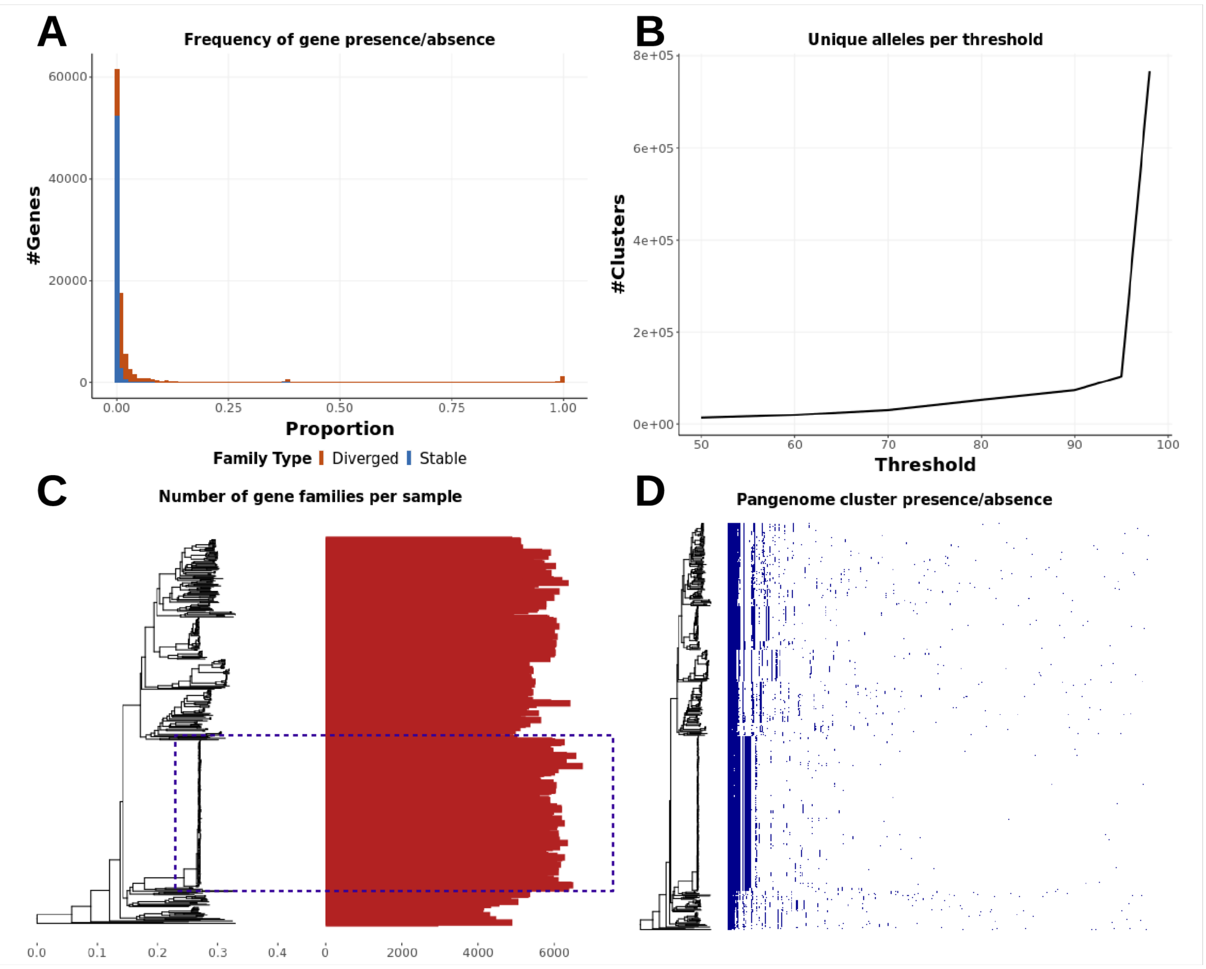

Figure 4. Summary figures of the pangenome of 496 *Pseudomonas* complete genomes. PIRATE was run on default parameters with an MCL inflation value of 6 and a HSP query length threshold of 0.9. (A) The proportion of genomes in which gene families are present. Gene families are considered stable (blue) when they have only a single allele at 98% amino acid identity, and diverged (red) when they have >1 allele. (B) The number of unique alleles at each amino acid percentage threshold. A unique allele is characterised as the highest percentage identity threshold at which a unique sub-cluster of isolates from a single gene family was identified by MCL. (C) The number of gene families per isolate ordered alongside the phylogenetic tree. (D) Shared gene presence per isolate ordered alongside the phylogenetic tree. Gene family presence is indicated by a blue block per column. Phylogenetic trees were generated from a core gene alignment from PIRATE and constructed using rapidnj [22].

Prochlorococcus marinus

PIRATE was applied to a dataset of 45 draft genomes of *Prochlorococcus marinus,* a marine cyanobacterium with extremely diverse gene complement, from the NCBI database (Supplementary Table 2) [21]. The pangenome of *Prochlorococcus marinus* was reconstructed, including gene sequence alignment, in 2,976s (50 min) using 8 threads, an MCL inflation value of 6 and a range of sequence similarity thresholds from 0-95 % (0,10,20,30,40,50,60,70,80,90 and 95 %). This relaxed range of sequence similarity thresholds allowed us to test the lower limits of BLAST/DIAMOND for detecting homology in these data. The pangenome comprised of 91,593 loci clustered into 8,325 gene clusters of which 867 (10.41 %) were considered core (present in >95% of isolates) (Figure 5.A). There were large number of genes present at intermediate frequency, most likely due to strong phylogeny structure within the limited sample size, and large numbers of genes private to related

lineages. The number of unique alleles per genome increased at percentage identities thresholds of >70 %, representing the inter-lineage divergence, and increased sharply at thresholds >94-95%, which is consistent with the sharp intra-species rise in allelic diversity observed in other species in this study (Figure 5.B). The majority of *Prochlorococcus marinus* isolates had a pangenome size of ~1800 genes per isolate with the exception of a single lineage which contained ~2600 genes (Figure 5.C). Interestingly, the additional genetic complement of this lineage was not comprised primarily of genes shared between all isolates, instead it contained a large proportion of rare genes (Figure 5.D). Observation of the number of shared genes alongside the core genome phylogenetic tree of *P. marinus* revealed that each of the deep branching lineages have a complement of approximately equal numbers of ‘lineage core’ genes (Figure 5.D).

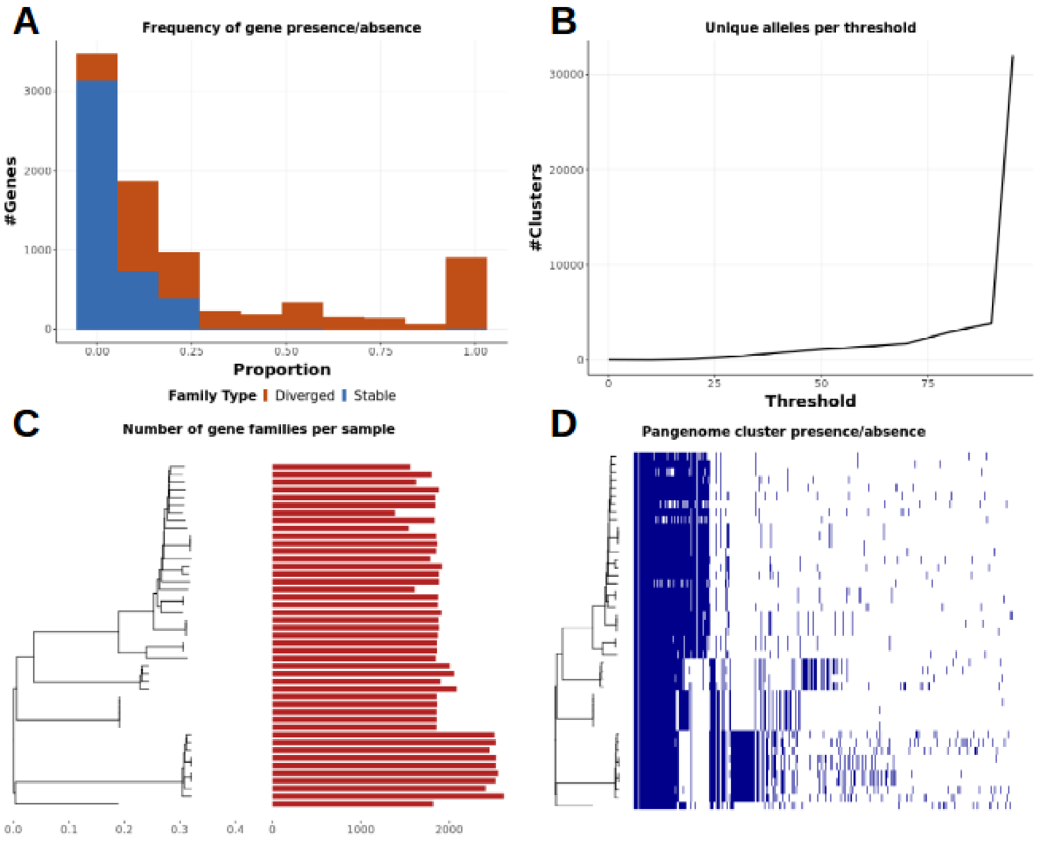

Figure 5. Summary figures of the pangenome of 45 *Prochlorococcus marinus* draft genomes. PIRATE was run on default parameters with an MCL inflation value of 6, and a HSP query length threshold of 0.9 and a sequence similarity step range of 0,10,20,30,40,50,60,70,80,90 and 95 %. (A) The proportion of genomes in which gene families are present. Gene families are considered stable (blue) when they have only a single allele at 98% amino acid identity, and diverged (red) when they have >1 allele. (B) The number of unique alleles at each amino acid percentage threshold. A unique allele is characterised as the highest percentage identity threshold at which a unique sub-cluster of isolates from a single gene family was identified by MCL. (C) The number of gene families per isolate ordered alongside the phylogenetic tree. (D) Shared gene presence per isolate ordered alongside the phylogenetic tree. Gene family presence is indicated by a blue block per column. Phylogenetic trees were generated from a core gene alignment from PIRATE and constructed using rapidnj [22].

Conclusion

Here we present PIRATE, a toolbox for pangenomic analysis of bacterial genomes, which provides a framework for exploring gene diversity by defining genes using relaxed sequence similarity thresholds. This pipeline builds upon existing tools using a novel methodology that can be applied to any annotated genomic features. PIRATE identifies and categorizes duplicated and disrupted genes, estimates allelic diversity, scores gene divergence and contextualizes genes using a pangenome graph. We demonstrate that it compares favourably with other commonly used tools for pangenomic analysis, in both execution time and computational resources, and is fully compatible with software for downstream analysis and visualisation. Furthermore, it is scalable to multiprocessor environments and can be applied to large numbers of genomes on modest hardware. Together the enhanced core and accessory genome characterisation capability, and the practical implementation advantages, make PIRATE a potentially powerful tool in bacterial genomics - a field in which there is an urgent need for tools that are applicable to increasingly large and complex datasets.

Acknowledgements

We would like to thank everyone who has contributed to the development of PIRATE through testing and feedback.

Funding

This work has been supported by BBSRC/NERC grant BB/M026388/1 awarded to E.F and MRC grant MR/L015080/1 awarded to S.S.

Conflict of Interest: none declared.

Authors Contributions

S.B. developed the software and wrote the manuscript. H.A.T. and N.M.C. contributed to and tested the software. S.K.S. and E.J.F. provided guidance and contributed to the manuscript.

Software Availability

Project name: "PIRATE: A fast and scalable pangenomics toolbox for clustering diverged orthologues in bacteria"
Project home page: https://github.com/SionBayliss/PIRATE
Operating system(s): Ubuntu 16.04, MacOS
Programming language: Perl, R.
Other requirements: mcl, mafft, cd-hit, fasttree, ncbi-blast+, bioperl, GNU parallel, diamond
License: GNU GPL v3.0
RRID: SCR_017265
Data Availability: An archival copy of the code, scripts and other supporting data are also available via the GigaScience database GigaDB [19].

References

1. Sheppard SK, Guttman DS, Fitzgerald JR. Population genomics of bacterial host adaptation. Nat Rev Genet. 2018;19:549–65.

2. Page AJ, Cummins CA, Hunt M, Wong VK, Reuter S, Holden MTG, et al. Roary: rapid large-scale prokaryote pan genome analysis. Bioinformatics. 2015;31:3691–3.

3. Thorpe HA, Bayliss SC, Sheppard SK, Feil EJ. Piggy: a rapid, large-scale pan-genome analysis tool for intergenic regions in bacteria. Gigascience. 2018;7:1–11.

4. Sahl JW, Caporaso JG, Rasko DA, Keim P. The large-scale blast score ratio (LS-BSR) pipeline: a method to rapidly compare genetic content between bacterial genomes. PeerJ. 2014;2:e332.

5. Li L, Stoeckert CJ Jr, Roos DS. OrthoMCL: identification of ortholog groups for eukaryotic genomes. Genome Res. 2003;13:2178–89.

6. Ding W, Baumdicker F, Neher RA. panX: pan-genome analysis and exploration. Nucleic Acids Res. 2018;46:e5.

7. Sheppard SK, Jolley KA, Maiden MCJ. A Gene-By-Gene Approach to Bacterial Population Genomics: Whole Genome MLST of Campylobacter. Genes . 2012;3:261–77.

8. Méric G, Yahara K, Mageiros L, Pascoe B, Maiden MCJ, Jolley KA, et al. A reference pan-genome approach to comparative bacterial genomics: identification of novel epidemiological markers in pathogenic Campylobacter. PLoS One. 2014;9:e92798.

9. Lees JA, Harris SR, Tonkin-Hill G, Gladstone RA, Lo SW, Weiser JN, et al. Fast and flexible bacterial genomic epidemiology with PopPUNK. Genome Res. 2019;29:304–16.

10. Denamur E, Matic I. Evolution of mutation rates in bacteria. Mol Microbiol. 2006;60:820–7.

11. Li W, Godzik A. Cd-hit: a fast program for clustering and comparing large sets of protein or nucleotide sequences. Bioinformatics. 2006;22:1658–9.

12. Buchfink B, Xie C, Huson DH. Fast and sensitive protein alignment using DIAMOND. Nat Methods. 2015;12:59–60.

13. Camacho C, Coulouris G, Avagyan V, Ma N, Papadopoulos J, Bealer K, et al. BLAST+: architecture and applications. BMC Bioinformatics. 2009;10:421.

14. Enright AJ, Van Dongen S, Ouzounis CA. An efficient algorithm for large-scale detection of protein families. Nucleic Acids Res. 2002;30:1575–84.

15. Katoh K, Misawa K, Kuma K-I, Miyata T. MAFFT: a novel method for rapid multiple sequence alignment based on fast Fourier transform. Nucleic Acids Res. 2002;30:3059–66.

16. Hadfield J, Croucher NJ, Goater RJ, Abudahab K, Aanensen DM, Harris SR. Phandango: an interactive viewer for bacterial population genomics. Bioinformatics. 2017.

17. Brynildsrud O, Bohlin J, Scheffer L, Eldholm V. Rapid scoring of genes in microbial pan-genome-wide association studies with Scoary. Genome Biol. 2016;17:238.

18. Argimón S, Abudahab K, Goater RJE, Fedosejev A, Bhai J, Glasner C, et al. Microreact: visualizing and sharing data for genomic epidemiology and phylogeography. Microb Genom. 2016;2:e000093.

19. Bayliss SC, Thorpe HA, Coyle NM, Sheppard SK, Feil EJ. Supporting data for “PIRATE: A fast and scalable pangenomics toolbox for clustering diverged orthologues in bacteria”. GigaScience Database. http://dx.doi.org/10.5524/100645

20. Connor TR, Loman NJ, Thompson S, Smith A, Southgate J, Poplawski R, et al. CLIMB (the Cloud Infrastructure for Microbial Bioinformatics): an online resource for the medical microbiology community. Microb Genom. 2016;2:e000086.

21. Pruitt KD, Tatusova T, Maglott DR. NCBI reference sequences (RefSeq): a curated non-redundant sequence database of genomes, transcripts and proteins. Nucleic Acids Res. 2007;35:D61–5.

22. Simonsen M, Mailund T, Pedersen CNS. Rapid Neighbour-Joining. Algorithms in Bioinformatics. Springer Berlin Heidelberg; 2008. p. 113–22.

Manuscript
Click here to access/download;Manuscript;Manuscript (2).docx
Click here to view linked References

Click here to access/download
Supplementary Material
Supplementary_Information.pdf

Supplementary Table 2

Click here to access/download
Supplementary Material
Supplementary_Table_2.tsv

Supplementary Table 3

Click here to access/download
Supplementary Material
Supplementary_Table_3.tsv

Personal Cover Letter
Click here to access/download;Personal Cover;cover_letter.pdf

Dr. Sion C. Bayliss
Milner Centre for Evolution
Department of Biology and Biochemistry
University of Bath
Claverton Down
Bath BA2 7AY
UK

29/07/2019
E-mail: s.bayliss@bath.ac.uk
Tel: +44 (0)7838 072372

Dear Editor,

Please find enclosed the manuscript titled 'PIRATE: A fast and scalable pangenomics toolbox for clustering diverged orthologues in bacteria', which we would like to be considered for publication as an Technical Note in Gigascience. In this manuscript we describe PIRATE, a software toolbox for pangenomic analysis of bacterial genomes, which provides a framework for exploring the high diversity of genes observed in bacteria. PIRATE uses a novel approach, assessing clusters over a range of sequence similarity thresholds, to define gene orthologues. The software, made freely available for download from Github, identifies and categorizes duplicated and disrupted genes, estimates allelic diversity, scores gene divergence and contextualizes genes using a pangenome graph. PIRATE builds upon existing tools, in both speed and scope, and provides novel and complementary features that can be applied to any annotated genomic feature. In this manuscript we describe the underlying method and demonstrate the utility using a reference collection of *Staphylococcus aureus* genomes, highlighting how the identification of divergent core genes leads to a more conservative estimate of pangenome size. We additionally apply PIRATE to other large and diverse datasets for the purposes of both benchmarking and in order to illustrate the potential applications of the software. Given the rapid technological advances in sequencing technology and the ever expanding number of genomes available from diverse species, PIRATE represents a timely application that will be of broad interest to researchers interested in the field of bacterial genomics.

Yours sincerely,
Dr Sion C. Bayliss, on behalf of all co-authors.
