# Supplementary material for: PIRATE: A fast and scalable pangenomics toolbox for clustering diverged orthologues in bacteria
Source: Gigascience. 2019 Oct 9;8(10):giz119. doi: 10.1093/gigascience/giz119 (PMC6785682; doi:10.1093/gigascience/giz119)
Supplement: giz119_Response_to_Reviewer_Comments_Original_Submission [file giz119_response_to_reviewer_comments_original_submission.pdf]

Author's Response To Reviewer Comments

Close

I would like to thank the editors for considering the manuscript for publication and the reviewers for their time and insightful contributions. I hope that the revisions detailed below contribute to an improved manuscript which will be of broad interest to researchers interested in the field of bacterial genomics.

Editor Notes:
Comment 1) Please register any new software application in the SciCrunch.org database to receive a RRID (Research Resource Identification Initiative ID) number, and include this in your manuscript (in the "software availability" section). This will facilitate tracking, reproducibility and re-use of your tool.

- “PIRATE is available as a software application in the SciCrunch.org database (RRID SCR_017265)” added to the ‘Software Availability’ section.

Reviewer #1: This is a well written paper describing a new pan-genome method called PIRATE. They compare it to the state of the art and provide many improvements, so it will be of great use to the microbial bioinformatics community. In particular they use Dimond to speed up comparisons, and do a much better job with paralogs and assembly errors compared to Roary (my tool). The software is easy to install via Conda, and it accepts a very commonly used annotation file format (GFF3 files from PROKKA), all things that are often overlooked in papers, so good work.

Comment 1) You should consider adding the GC content of the 3 species under test since there is a nice range.

- Line 152 was amended to “Three bacterial species were selected for comparison, Campylobacter jejuni, Staphylococcus aureus and Escherichia coli, representing both a range of pangenome sizes (small, medium and large respectively) and GC contents (30.4%, 32.7% and 50.6% respectively)(Supplementary Table 2)”.

Comment 2) PopPunk (Lees et al.) has recently come out and would be a relevant citation:

- PopPunk has been added as a citation in the introduction. Additionally Line 252 was changed to “The large increase in the size of the accessory genome content inferred using Roary is primarily due to the post-processing (paralog splitting) of accessory genes and has also been described in previous studies [9].”

Comment 3) Could you add the inflation factor for MCL and how you arrived at it, because it can have a big impact on the end results.

- This was an important parameter for which the inclusion the default parameter was overlooked in the original manuscript. The following text was added to the Methods section, Line 89 - “A default MCL inflation value of 2 was identified as appropriate for intra-species clustering by this study and previous authors . A larger inflation value maybe appropriate for inter-species comparisons and can be modified within the software.”.

Reviewer #2: This manuscript presents the pangenomic analysis pipeline PIRATE, benchmarks it and

compares it to other tools roary and panX (I am one of the developers of panX). The tool implements a series of sensible steps to cluster annotated features into orthologous groups.

Comment 1) Benchmarking is a little underwhelming. The tool is marketed as being able to deal with genome collection at many different degrees of divergence and diversity. Testing on just 253 Staph aureus genomes doesn't really do this justice, the results for E coli and Campy are restricted to performance in the supplement. Why not also test on very diverse species (like Plochlorococcus marinus) or entire orders such as Pseudomonadales. The comparisons between tools are also restricted to two numbers (core and accessory genes). More informative comparisons would be between cluster size distribution and some analysis of whether the different tools actually found the same clusters.

- We thank the reviewer for this comment and have applied PIRATE to two additional example datasets, 48 draft genomes of Prochlorococcus marinus and a collection of 497 complete genomes of Pseudomonas species. For brevity the results of these analyses are included in the 'Additional Examples' section of the Supplementary Analysis (Supplementary Figures 7+8) and the presence of these additional examples have been alluded to in the 'Application to Real Data' section of the main text at Line 273 - "Additional examples of real data processed using PIRATE have been included in the Supplementary Analysis to highlight application of the tool to large or diverse datasets (Supplementary Figures 7+8). PIRATE was applied to 48 draft genomes of Prochlorococcus marinus, a marine cyanobacteria with extremely diverse gene complement, and a collection of 497 complete genomes of assorted Pseudomonas species, a genus of Gram-negative Gammaproteobacteria with highly variable genome sizes.".

In addition to this we have performed some additional clustering analysis which has been detailed in the Supplementary Analysis (Section: , Figure 9). The text "An analysis of the clusters produced by the tools indicated that there was broad intersection between metholodogies when considering core genes, but that differences become more pronounced in the intermediate and accessory pangenome (Supplementary Analysis, Supplementary Figure 9)." was added at Line 255 to address the relevant finding in the main text.

Comment 2) Page 1, last paragraph: The issue of overclustering/underclustering should be discussed a little more. In particular, I think the authors should highlight the fact that there is no objective truth to compare against and what is considered a useful clustering output to some extent depends on the downstream analysis.

- Many thanks to the reviewer for this excellent comment, this is a very relevant point which improves the readers' understanding of the scope of the work. The following lines have been added to the introduction at Line 54 - "The impact of over- and under-clustering is relevant to consider in the context of downstream research applications. Under-clustering (or over-splitting) can create a misleading impression of pangenome diversity and composition when considering how much gene diversity exists in the accessory genome [9]. However, for a study identifying genetic determinants associated with a phenotype, such as antibiotic resistance, core and accessory allelic variation which has been misclassified as additional accessory genes may have little to no impact as the causative genes in question may still be still correctly identified."

Comment 3) PanX runtimes are quadratic due to all against all comparison. But in the divide and conquer mode, runtimes are linear other than for the tree building step. Using runtimes when running panX with `-dmdc` would be a more appropriate comparison.

- In order to make the methodological comparison more appropriate the benchmarking of PanX was rerun using -dmdc and –subset_size (set to #samples/#threads). The results do not substantially change the comparisons between the tools but the execution time of PanX is significantly reduced. The results have been updated in the relevant panels in Figure 2 and the text in the main manuscript amended:

a) Line 174 added "In order to aid comparison PanX was used with the -dmdc option which allows multithreading of DIAMOND. Without this option the run time of PanX scales quadratically and is inappropriate for larger datasets and comparison to the other tools."

b) The paragraph following Figure 2 (Line 181) has been slightly amended to more clearly delineate between PIRATE using BLAST or DIAMOND. It now reads:
“The execution time of Roary and PIRATE scaled in an approximately linear manner with increasing number of samples (Figure 2.A). PanX scaled super-linearly, making application to larger datasets potentially problematic. Roary and PIRATE were faster than PanX at all time points without gene-by-gene alignment. The execution time of PIRATE using DIAMOND was comparable to that of Roary without gene-by gene alignment (Figure 2.A, top panel). Roary completed marginally quicker than PIRATE using BLAST without gene-by-gene alignment at all sample sizes. When gene-by-gene alignment was applied both Roary and PIRATE scaled sub-linearly with number of samples, however PIRATE using DIAMOND or BLAST completed substantially faster than either Roary or PanX (Figure 2.A, bottom panel). PIRATE exhibited lower memory usage than the other tools tested, scaling sub-linearly with number of samples (Figure 2.B). In conclusion, PIRATE compared favourably in both execution time and memory usage and these metrics suggest PIRATE can be flexibly applied to large datasets on routinely available hardware”

Comment 4) On page 4, line 5, you state that panX requires an alignment for paralog splitting. This is correct. But it also requires a tree. This is where the main computational overhead comes from.

- Line 169 has been amended to “It should be noted that both PIRATE and Roary include post-processing of paralogs in the comparison without alignment or phylogentic tree reconstruction, producing a complete output. PanX does not do this, as alignment, followed by tree building, is a necessary step in paralog identification in this pipeline. ”

Comment 5) Fig 3D is rather unhelpful and difficult to parse in its current form. All relevant parse happen in a tiny fraction of the figure and the 0-line for the upper part has to be guessed.

- Figure 3D has been amended with rescaled axis in order to provide more space for the relevant information and to provide an easily observed zero value on the x-axis of the top panel.

Comment 6) After some fiddling, I could get the pipeline to work. See problems I encountered below.

- I have updated the installation instructions (github README) to specify that the bioconda channels should be added before installing PIRATE in order to clear up any confusion (below):

conda config --add channels defaults
conda config --add channels bioconda
conda config --add channels conda-forge

Reviewer #3: PIRATE represents an interesting method to conduct pan-genomics by comparing the number of clusters at different clustering thresholds. I installed the software easily through CONDA and it seemed to work well on the datasets that I tested.

Comment 1) Reading the paper, I would have liked to see further delineation between PIRATE and other tools. For example, what are the biological ramifications of large cluster sizes at lower identities? I realize that this paper really discusses the method and not the applications, but some application would be helpful on how different clustering thresholds affect the interpretation.

We have expanded upon the examples as suggested by the referee (Line 209):

“PIRATE can quickly be used to identify genes with both highly conserved or divergent sequence similarity or variable copy number. The biological ramifications of these genes will vary between applications. A number of the genes exhibiting high amino acid sequence divergence have been well studied. For example the the core ‘accessory regulator’ agr locus exhibited a range of sequence identity clustering thresholds; agrA clusters at 91 %, agrB and agrC at 65 % and agrD at 45 % amino acid identity, each with a copy number of 1. We identified that another gene, ArlR, which is known to interact with the agr locus, has a similarly low amino acid similarity of 45 % perhaps implying that the linked genes have undergone similarly patterns of diversifying selection. This example highlights how

diversification may lead to over-splitting of genes if only a single sequence identity threshold were used, even if this threshold were applicable to the vast majority of genes in the pangenome. Expansion of families of MGEs or individual genes within the population can also be identified from the outputs. For example, IS256, known to play a role in biofilm formation and resistance to various antimicrobials, is present in 35 genomes, has a conserved amino acid sequence (<2% divergence) but a variable copy number of between 1 to 32 copies within the genomes in which it is present. Using these data is is possible to identify the strains which have an increased dosage of IS256."

Comment 2) I did have some questions about the time to run PIRATE. The manuscript suggests that it is faster than Roary using either blast or diamond. When I run Roary and PIRATE on your set of 100 E. coli genomes using default parameters and 8 processors, I find that Roary finished in 21m46s and PIRATE finished in 1h14m.

- The run time of PIRATE is faster than roary when alignment (roary: -en, PIRATE: -a) is toggled on. This is because alignment is fully parallelized in PIRATE. PIRATE running on default parameters will not be faster than roary without alignment as PIRATE follows many of the steps in the roary pipeline, plus the addition of multiple MCL thresholds followed by paralog identification and classification (which is more computationally expensive than the paralog splitting of roary due to the use of CDHIT and BLAST on a per cluster basis). To more explicitly address this point Line 184 was modified to "The execution time of PIRATE using DIAMOND was comparable to that of Roary without gene-by gene alignment (Figure 2.A, top panel). Roary completed marginally faster than PIRATE using BLAST without gene-by-gene alignment at all sample sizes." and Line 186 to "When gene-by-gene alignment was applied both Roary and PIRATE scaled sub-linearly with number of samples, however PIRATE completed substantially faster than Roary and PanX (Figure 2.A, bottom panel)."

Comment 3) There may also be some issues scaling with genome diversity. For example, running PIRATE on 61 Orientia tsutsugamushi genomes with default PIRATE parameters, took over 4 hours to complete: "PIRATE completed in 14803s". This makes me worry about the scalability of the algorithm to larger, complex datasets. I think that additional benchmarking on large and complex datasets would help convince me that this method will scale with increasingly large datasets.

- The time to complete PIRATE scales linearly with sample size. Large numbers of paralogous genes, either real or caused by poor assemblies, will increase run time. For the purpose of the current manuscript all samples have been run on default settings. This may be suboptimal for more diverse datasets. There are various options available to reduce the potential runtime of PIRATE for large or diverse datasets, such as excluding HSPs that are not below a set proportion of the query length, increasing the MCL inflation value to reduce over-clustering (and therefore reduce paralogs) or by using DIAMOND as a faster alternative to BLAST. In order to address these concerns I have run PIRATE on two additional diverse collections of bacterial genomes using some of the options to reduce execution time. These included 497 complete genomes of the genus Pseudomonas that were available from the RefSeq database and a dataset of 48 Prochlorococcus marinus draft genomes, a diverse bacterial species suggested by Reviewer 1. Prochlorococcus marinus was used in preference to Orientia tsutsugamushi due to a larger number of publicly available genomes with a similarly large and diverse accessory genome. PIRATE completed in 2976s (50 mins) for the Prochlorococcus marinus dataset and 188,216s (52.3h) for the larger Pseudomonas dataset. We believe that these run times are consistent with the application of PIRATE to large and/or diverse datasets using accessible hardware. Additional results have been added in the 'Additional Examples' section of the Supplementary Analysis (Supplementary Figures 7+8).

Close
