# Supplementary material for: PIRATE: A fast and scalable pangenomics toolbox for clustering diverged orthologues in bacteria
Source: Gigascience. 2019 Oct 9;8(10):giz119. doi: 10.1093/gigascience/giz119 (PMC6785682; doi:10.1093/gigascience/giz119)
Supplement: giz119_Response_to_Reviewer_Comments_Revision_1 [file giz119_response_to_reviewer_comments_revision_1.pdf]

Author's Response To Reviewer Comments

Close

I would like to thank the editors for considering the manuscript for publication and the reviewers for their time and insightful contributions.

General Notes:

- A software availability section was added to the end of the manuscript (before References).
- The accession numbers for all isolates in the main and supplementary text are included in Supplementary Table 2.

Editor's Note - I agree with reviewer 2 that the additional tests and benchmarks with more complex datasets, included during the revision in the supplement, should be moved to the main manuscript.

- The sections of the supplementary materials explicitly mentioned above have been moved to the main manuscript. The two supplementary sections entitled 'Procholoccocus marinus' and 'Pseudomonas' were inserted after the 'Application to real datasets' section along with the relevant figures. A short foreword was added to the section 'Application to real datasets' to improve the flow of the manuscript. The section 'Cluster Comparison Between Pangenome Tools' has been incorporated into 'Application to real data' (Staphylococcus aureus) section as a separate paragraph enlarging on the clustering comparison already present in the main text. The relevant figure has remained in the supplementary materials. Minor changes to the text in have been made these sections in order to keep the manuscript concise and to remove any redundancy within the revised text.

Reviewer #2: The authors have revised their manuscript and addressed most points during the review. My preference would be to include the additional tests and benchmarks in the main text, but this is up to the authors and editor. The explicit comparison between clusters seems to have revealed that that panX and Pirate find mostly the same clusters, while PIRATE splits accessory genes more aggressively. The Prochlorococcus suggests that PIRATE has a tendency to break up core gene clusters (PIRATE finds 651 core genes -- this should probably be about twice as much. This is also quite apparent in Fig S9.D where each core genome cluster has about 500 'private' genes which likely do have homologous partners in the other groups.). I think there is more that could be done here, but as a technical report that describes the software, the manuscript is sufficient in my opinion.

As suggested by reviewer 2 and in agreement with the editor's comment (see above) the additional benchmarking analyses performed during the previous revision has been moved into the main manuscript. Relevant text, figures, legends and references have been updated to accommodate this change.

In order to address the points raised by the reviewer pertaining to the results of the Prochlorococcus analysis we updated the analysis using an expanded range of sequence identity thresholds between 0% (i.e. no thresholding based upon sequence similarity) and 95%. This made little difference to the results of the analysis. This relaxed range of sequence similarity thresholds allowed us to test the lower limits of BLAST/DIAMOND for detecting homology in these data. The updated analysis increases the number of core genes identified (650→867 genes) but it does not remove the presence of the 'lineage specific' genes that were observed previously. Whilst this does not preclude the possibility that these genes have undetected homologous partners within the rest of the dataset it does suggest that this level of homology is undetectable using the suite of sequence homology methodologies shared by the pangenome tools under comparison in the current manuscript. Alternative methodologies able to detect deeper sequence homology, such as HMMs, may be more suitable for investigating this further, but the application of these methodologies lies outside of the purview of the current manuscript. The updated

analysis was incorporated into the main text. Minor changes have been made to the text to reflect the differences in the size estimates between the two analyses.

1/ The discussion of the panX flat -dmdc is not accurate. DIAMOND uses multiple cores even without that flag (provided the -t flag is used to specify the number of available CPUs). The dmdc flag results in splitting of the pangenome into batches followed by merging of the pangenomes of these batches.

Line 175 was amended to read "In order to aid comparison PanX was used with the -dmdc flag which batches input genomes, clusters per batch and subsequently merges the batches."

2/ panX has been applied to data sets in excess of 2000 strains and the comment panX's applicability to large data sets unnecessary -- in particular as the biggest data sets you test contain at most 500 sequences. The n^3/2 scaling is not really that critical. Furthermore, this is entirely due to tree building step. This enables the panX visualization of gene trees and inference of mutational events -- features the other tools don't offer.

The text "PanX scaled super-linearly, making application to larger datasets potentially problematic." was removed at line 183.

3/ line 269: "low homology thresholds". I would rephrase this as "low identity threshold"

The modification was made at Line 269.

4/ many figures have tiny labels.

The figures in the main text have been amended to have larger font sizes.

5/ supplement, Prochlorococcus: I am unsure what you mean by "pangenome size of an isolate" (Fig 8C and the text referring to it). This really is more like "number of genes" (corrected for recent duplications).

The relevant text has been modified throughout the paragraph and associated figure legend.

6/ accession numbers of the additional data sets should be added to the supplementary tables

The accession numbers for all isolate in the main and supplementary text are included in Supplementary Table 2.

7/ explicit documentation of the options given to the different tools would help (a file with the commands for pirate, roary and panX).

The following text was added at Line 155 "The scripts used to perform these analyses are available from the GigaDB repository associated with this publication [19]. The settings used for each tool have been detailed in Supplementary Table 3.". Supplementary Table 3 was added. It contains the settings for the various tools used for the benchmarking analyses.

Reviewer #3: The authors have addressed all of my questions/concerns

Close
