# Supplementary material for: PIRATE: A fast and scalable pangenomics toolbox for clustering diverged orthologues in bacteria
Source: Gigascience. 2019 Oct 9;8(10):giz119. doi: 10.1093/gigascience/giz119 (PMC6785682; doi:10.1093/gigascience/giz119)
Supplement: giz119_Response_to_Reviewer_Comments_Revision_2 [file giz119_response_to_reviewer_comments_revision_2.pdf]

Author's Response To Reviewer Comments

Close

The manuscript has been attached as a .docx formatted file. PDF also attached.

Editor Notes:

1) Please add the GigaDB DOI link to your reference [19] in the bibliography (http://dx.doi.org/10.5524/100645):
Added to reference.

2) Please add a sentence at the end of your "software availability" section along the lines of: "An archival copy of the code, scripts and other supporting data are also available via the GigaScience database GigaDB [19]."
Added in text.

Close
