# Supplementary material for: PIRATE: A fast and scalable pangenomics toolbox for clustering diverged orthologues in bacteria
Source: Gigascience. 2019 Oct 9;8(10):giz119. doi: 10.1093/gigascience/giz119 (PMC6785682; doi:10.1093/gigascience/giz119)
Supplement: giz119_Reviewer_1_Report_Original_Submission — Andrew Page -- 5/7/2019 Reviewed [file giz119_reviewer_1_report_original_submission.pdf]

**Reviewer Report**

**Title: PIRATE: A fast and scalable pangenomics toolbox for clustering diverged orthologues in bacteria**

**Version: Original Submission** **Date:** 5/7/2019

**Reviewer name: Andrew Page**

**Reviewer Comments to Author:**

This is a well written paper describing a new pan-genome method called PIRATE.  They compare it to the state of the art and provide many improvements, so it will be of great use to the microbial bioinformatics community.  In particular they use Dimond to speed up comparisons, and do a much better job with paralogs and assembly errors compared to Roary (my tool).  The software is easy to install via Conda, and it accepts a very commonly used annotation file format (GFF3 files from PROKKA), all things that are often overlooked in papers, so good work.
Just a few minor comments:
You should consider adding the GC content of the 3 species under test since there is a nice range.
PopPunk (Lees et al.) has recently come out and would be a relevant citation:
https://genome.cshlp.org/content/29/2/304
Could you add the inflation factor for MCL and how you arrived at it, because it can have a big impact on the end results.

**Level of Interest**

Please indicate how interesting you found the manuscript: Choose an item.

**Quality of Written English**

Please indicate the quality of language in the manuscript: Choose an item.

**Declaration of Competing Interests**

Please complete a declaration of competing interests, considering the following questions:

- Have you in the past five years received reimbursements, fees, funding, or salary from an organisation that may in any way gain or lose financially from the publication of this manuscript, either now or in the future?
- Do you hold any stocks or shares in an organisation that may in any way gain or lose financially from the publication of this manuscript, either now or in the future?
- Do you hold or are you currently applying for any patents relating to the content of the manuscript?
- Have you received reimbursements, fees, funding, or salary from an organization that holds or has applied for patents relating to the content of the manuscript?

- Do you have any other financial competing interests?
- Do you have any non-financial competing interests in relation to this paper?

If you can answer no to all of the above, write 'I declare that I have no competing interests' below. If your reply is yes to any, please give details below.

I declare that I have no competing interests

I agree to the open peer review policy of the journal. I understand that my name will be included on my report to the authors and, if the manuscript is accepted for publication, my named report including any attachments I upload will be posted on the website along with the authors' responses. I agree for my report to be made available under an Open Access Creative Commons CC-BY license (http://creativecommons.org/licenses/by/4.0/). I understand that any comments which I do not wish to be included in my named report can be included as confidential comments to the editors, which will not be published.

Choose an item.

To further support our reviewers, we have joined with Publons, where you can gain additional credit to further highlight your hard work (see: https://publons.com/journal/530/gigascience). On publication of this paper, your review will be automatically added to Publons, you can then choose whether or not to claim your Publons credit. I understand this statement.

Yes Choose an item.
