# Supplementary material for: PIRATE: A fast and scalable pangenomics toolbox for clustering diverged orthologues in bacteria
Source: Gigascience. 2019 Oct 9;8(10):giz119. doi: 10.1093/gigascience/giz119 (PMC6785682; doi:10.1093/gigascience/giz119)
Supplement: giz119_Reviewer_2_Report_Original_Submission — Richard Neher -- 5/11/2019 Reviewed [file giz119_reviewer_2_report_original_submission.pdf]

Reviewer Report

Title: PIRATE: A fast and scalable pangenomics toolbox for clustering diverged orthologues in bacteria

Version: Original Submission **Date:** 5/11/2019

Reviewer name: Richard Neher

Reviewer Comments to Author:

This manuscript presents the pangenomic analysis pipeline PIRATE, benchmarks it and compares it to other tools roary and panX (I am one of the developers of panX). The tool implements a series of sensible steps to cluster annotated features into orthologous groups.

- benchmarking is a little underwhelming. The tool is marketed as being able to deal with genome collection at many different degrees of divergence and diversity. Testing on just 253 Staph aureus genomes doesn't really do this justice, the results for E coli and Campy are restricted to performance in the supplement. Why not also test on very diverse species (like Plochlorococcus marinus) or entire orders such as Pseudomonadales. The comparisons between tools are also restricted to two numbers (core and accessory genes). More informative comparisons would be between cluster size distribution and some analysis of whether the different tools actually found the same clusters.
- page 1, last paragraph: The issue of overclustering/underclustering should be discussed a little more. In particular, I think the authors should highlight the fact that there is no objective truth to compare against and what is considered a useful clustering output to some extent depends on the downstream analysis.
- panX runtimes are quadratic due to all against all comparison. But in the divide and conquer mode, runtimes are linear other than for the tree building step. Using runtimes when running panX with `-dmdc` would be a more appropriate comparison.
- on page 4, line 5, you state that panX requires an alignment for paralog splitting. This is correct. But it also requires a tree. This is where the main computational overhead comes from.
- fig 3D is rather unhelpful and difficult to parse in its current form. All relevant parse happen in a tiny fraction of the figure and the 0-line for the upper part has to be guessed.
- After some fiddling, I could get the pipeline to work. See problems I encountered below.

Installation notes:

Conda installation failed:

> \:> conda --version
> conda 4.6.11
> ran13:/home/richard/Private/Refereeing/2019-05_Giga
> \:> conda install -c sionbayliss pirate
> WARNING: The conda.compat module is deprecated and will be removed in a future release.
> Collecting package metadata: done
> Solving environment: failed
>
> PackagesNotFoundError: The following packages are not available from current channels:

>
>   - pirate -> blast[version='>=2.2.31']
>   - pirate -> cd-hit[version='>=4.6.4']
>   - pirate -> diamond[version='>=0.9.14']
>   - pirate -> fasttree[version='>=2.1.10']
>   - pirate -> mafft==7.310
>   - pirate -> mcl[version='>=14.137']
>   - pirate -> parallel[version='>=20170422']
>   - pirate -> perl-bioperl==1.7.2
>
> Current channels:
>
>   - https://conda.anaconda.org/sionbayliss/linux-64
>   - https://conda.anaconda.org/sionbayliss/noarch
>   - https://repo.anaconda.com/pkgs/main/linux-64
>   - https://repo.anaconda.com/pkgs/main/noarch
>   - https://repo.anaconda.com/pkgs/free/linux-64
>   - https://repo.anaconda.com/pkgs/free/noarch
>   - https://repo.anaconda.com/pkgs/r/linux-64
>   - https://repo.anaconda.com/pkgs/r/noarch
>
> To search for alternate channels that may provide the conda package you're
> looking for, navigate to
>
>   https://anaconda.org
>
> and use the search bar at the top of the page.
Adding `-c bioconda` fixes most dependencies, but not `parallel`

Level of Interest

Please indicate how interesting you found the manuscript: Choose an item.

Quality of Written English

Please indicate the quality of language in the manuscript: Choose an item.

Declaration of Competing Interests

Please complete a declaration of competing interests, considering the following questions:

- Have you in the past five years received reimbursements, fees, funding, or salary from an organisation that may in any way gain or lose financially from the publication of this manuscript, either now or in the future?
- Do you hold any stocks or shares in an organisation that may in any way gain or lose financially from the publication of this manuscript, either now or in the future?
- Do you hold or are you currently applying for any patents relating to the content of the manuscript?
- Have you received reimbursements, fees, funding, or salary from an organization that holds or has applied for patents relating to the content of the manuscript?
- Do you have any other financial competing interests?
- Do you have any non-financial competing interests in relation to this paper?

If you can answer no to all of the above, write 'I declare that I have no competing interests' below. If your reply is yes to any, please give details below.

none

I agree to the open peer review policy of the journal. I understand that my name will be included on my report to the authors and, if the manuscript is accepted for publication, my named report including any attachments I upload will be posted on the website along with the authors' responses. I agree for my report to be made available under an Open Access Creative Commons CC-BY license (http://creativecommons.org/licenses/by/4.0/). I understand that any comments which I do not wish to be included in my named report can be included as confidential comments to the editors, which will not be published.

Choose an item.

To further support our reviewers, we have joined with Publons, where you can gain additional credit to further highlight your hard work (see: https://publons.com/journal/530/gigascience). On publication of this paper, your review will be automatically added to Publons, you can then choose whether or not to claim your Publons credit. I understand this statement.

Yes Choose an item.
