# Supplementary material for: PIRATE: A fast and scalable pangenomics toolbox for clustering diverged orthologues in bacteria
Source: Gigascience. 2019 Oct 9;8(10):giz119. doi: 10.1093/gigascience/giz119 (PMC6785682; doi:10.1093/gigascience/giz119)
Supplement: giz119_Reviewer_2_Report_Revision_1 — Richard Neher -- 8/7/2019 Reviewed [file giz119_reviewer_2_report_revision_1.pdf]

Reviewer Report

Title: PIRATE: A fast and scalable pangenomics toolbox for clustering diverged orthologues in bacteria

Version: Revision 1 **Date:** 8/7/2019

Reviewer name: Richard Neher

Reviewer Comments to Author:

The authors have revised their manuscript and addressed most points during the review. My preference would be to include the additional tests and benchmarks in the main text, but this is up to the authors and editor. The explicit comparison between clusters seems to have revealed that that panX and Pirate find mostly the same clusters, while PIRATE splits accessory genes more aggressively. The Prochlorococcus suggests that PIRATE has a tendency to break up core gene clusters (PIRATE finds 651 core genes -- this should probably be about twice as much. This is also quite apparent in Fig S9.D where each core genome cluster has about 500 'private' genes which likely do have homologous partners in the other groups.). I think there is more that could be done here, but as a technical report that describes the software, the manuscript is sufficient in my opinion.

A few additional issues.

- The discussion of the panX flat -dmdc is not accurate. DIAMOND uses multiple cores even without that flag (provided the -t flag is used to specify the number of available CPUs). The dmdc flag results in splitting of the pangenome into batches followed by merging of the pangenomes of these batches.
- panX has been applied to data sets in excess of 2000 strains and the comment panX's applicability to large data sets unnecessary -- in particular as the biggest data sets you test contain at most 500 sequences. The n^3/2 scaling is not really that critical. Furthermore, this is entirely due to tree building step. This enables the panX visualization of gene trees and inference of mutational events -- features the other tools don't offer.
- line 269: "low homology thresholds". I would rephrase this as "low identity threshold"
- many figures have tiny labels.
- supplement, Prochlorococcus: I am unsure what you mean by "pangenome size of an isolate" (Fig 8C and the text referring to it). This really is more like "number of genes" (corrected for recent duplications).
- accession numbers of the additional data sets should be added to the supplementary tables
- explicit documentation of the options given the to different tools would help (a file with the commands for pirate, roary and panX).

Level of Interest

Please indicate how interesting you found the manuscript: Choose an item.

Quality of Written English

Please indicate the quality of language in the manuscript: Choose an item.

Declaration of Competing Interests

Please complete a declaration of competing interests, considering the following questions:

- Have you in the past five years received reimbursements, fees, funding, or salary from an organisation that may in any way gain or lose financially from the publication of this manuscript, either now or in the future?
- Do you hold any stocks or shares in an organisation that may in any way gain or lose financially from the publication of this manuscript, either now or in the future?
- Do you hold or are you currently applying for any patents relating to the content of the manuscript?
- Have you received reimbursements, fees, funding, or salary from an organization that holds or has applied for patents relating to the content of the manuscript?
- Do you have any other financial competing interests?
- Do you have any non-financial competing interests in relation to this paper?

If you can answer no to all of the above, write 'I declare that I have no competing interests' below. If your reply is yes to any, please give details below.

I declare that I have no competing interests

I agree to the open peer review policy of the journal. I understand that my name will be included on my report to the authors and, if the manuscript is accepted for publication, my named report including any attachments I upload will be posted on the website along with the authors' responses. I agree for my report to be made available under an Open Access Creative Commons CC-BY license (http://creativecommons.org/licenses/by/4.0/). I understand that any comments which I do not wish to be included in my named report can be included as confidential comments to the editors, which will not be published.

Choose an item.

To further support our reviewers, we have joined with Publons, where you can gain additional credit to further highlight your hard work (see: https://publons.com/journal/530/gigascience). On publication of this paper, your review will be automatically added to Publons, you can then choose whether or not to claim your Publons credit. I understand this statement.

Yes Choose an item.
