# Supplementary material for: PIRATE: A fast and scalable pangenomics toolbox for clustering diverged orthologues in bacteria
Source: Gigascience. 2019 Oct 9;8(10):giz119. doi: 10.1093/gigascience/giz119 (PMC6785682; doi:10.1093/gigascience/giz119)
Supplement: giz119_Reviewer_3_Report_Original_Submission — Jason Sahl -- 5/16/2019 Reviewed [file giz119_reviewer_3_report_original_submission.pdf]

Reviewer Report

Title: PIRATE: A fast and scalable pangenomics toolbox for clustering diverged orthologues in bacteria

Version: Original Submission **Date:** 5/16/2019

Reviewer name: Jason Sahl

Reviewer Comments to Author:

PIRATE represents an interesting method to conduct pan-genomics by comparing the number of clusters at different clustering thresholds. I installed the software easily through CONDA and it seemed to work well on the datasets that I tested.

Reading the paper, I would have liked to see further delineation between PIRATE and other tools. For example, what are the biological ramifications of large cluster sizes at lower identities? I realize that this paper really discusses the method and not the applications, but some application would be helpful on how different clustering thresholds affect the interpretation.

I did have some questions about the time to run PIRATE. The manuscript suggests that it is faster than Roary using either blast or diamond. When I run Roary and PIRATE on your set of 100 E. coli genomes using default parameters and 8 processors, I find that Roary finished in 21m46s and PIRATE finished in 1h14m.

My commands:

roary -p 8 gffs/*gff

PIRATE -i gffs/ -t 8

There may also be some issues scaling with genome diversity. For example, running PIRATE on 61 Orientia tsutsugamushi genomes with default PIRATE parameters, took over 4 hours to complete: "PIRATE completed in 14803s". This makes me worry about the scalability of the algorithm to larger, complex datasets. I think that additional benchmarking on large and complex datasets would help convince me that this method will scale with increasingly large datasets.

Level of Interest

Please indicate how interesting you found the manuscript: Choose an item.

Quality of Written English

Please indicate the quality of language in the manuscript: Choose an item.

Declaration of Competing Interests

Please complete a declaration of competing interests, considering the following questions:

- Have you in the past five years received reimbursements, fees, funding, or salary from an organisation that may in any way gain or lose financially from the publication of this manuscript, either now or in the future?
- Do you hold any stocks or shares in an organisation that may in any way gain or lose financially from the publication of this manuscript, either now or in the future?
- Do you hold or are you currently applying for any patents relating to the content of the manuscript?
- Have you received reimbursements, fees, funding, or salary from an organization that holds or has applied for patents relating to the content of the manuscript?
- Do you have any other financial competing interests?
- Do you have any non-financial competing interests in relation to this paper?

If you can answer no to all of the above, write 'I declare that I have no competing interests' below. If your reply is yes to any, please give details below.

I declare that I have no competing interests

I agree to the open peer review policy of the journal. I understand that my name will be included on my report to the authors and, if the manuscript is accepted for publication, my named report including any attachments I upload will be posted on the website along with the authors' responses. I agree for my report to be made available under an Open Access Creative Commons CC-BY license (http://creativecommons.org/licenses/by/4.0/). I understand that any comments which I do not wish to be included in my named report can be included as confidential comments to the editors, which will not be published.

Choose an item.

To further support our reviewers, we have joined with Publons, where you can gain additional credit to further highlight your hard work (see: https://publons.com/journal/530/gigascience). On publication of this paper, your review will be automatically added to Publons, you can then choose whether or not to claim your Publons credit. I understand this statement.

Yes Choose an item.
